# A novel prostaglandin E receptor 4 (EP4) small molecule antagonist induces articular cartilage regeneration

**DOI:** 10.1038/s41421-022-00382-6

**Published:** 2022-03-08

**Authors:** Yunyun Jin, Qianqian Liu, Peng Chen, Siyuan Zhao, Wenhao Jiang, Fanhua Wang, Peng Li, Yuanjin Zhang, Weiqiang Lu, Tao P. Zhong, Xinran Ma, Xin Wang, Alison Gartland, Ning Wang, Karan Mehul Shah, Hankun Zhang, Xu Cao, Lei Yang, Mingyao Liu, Jian Luo

**Affiliations:** 1 Shanghai Key Laboratory of Regulatory Biology, Institute of Biomedical Sciences and School of Life Sciences, East China Normal University, Shanghai, China; 2 Yangzhi Rehabilitation Hospital (Sunshine Rehabilitation Centre), Tongji University School of Medicine, Shanghai, China; 3 Orthopedic Institute, Soochow University, Suzhou, Jiangsu China; 4 Department of Oncology and Metabolism, The University of Sheffield, Sheffield, UK; 5 Departments of Orthopaedic Surgery and Biomedical Engineering and Institute of Cell Engineering, The Johns Hopkins University School of Medicine, Baltimore, MD USA; 6 Center for Health Science and Engineering, School of Materials Science and Engineering, Hebei University of Technology, Tianjin, China

**Keywords:** Mechanisms of disease, Cell signalling

## Abstract

Articular cartilage repair and regeneration is an unmet clinical need because of the poor self-regeneration capacity of the tissue. In this study, we found that the expression of prostaglandin E receptor 4 (PTGER4 or EP4) was largely increased in the injured articular cartilage in both humans and mice. In microfracture (MF) surgery-induced cartilage defect (CD) and destabilization of the medial meniscus (DMM) surgery-induced CD mouse models, cartilage-specific deletion of *EP4* remarkably promoted tissue regeneration by enhancing chondrogenesis and cartilage anabolism, and suppressing cartilage catabolism and hypertrophy. Importantly, knocking out *EP4* in cartilage enhanced stable mature articular cartilage formation instead of fibrocartilage, and reduced joint pain. In addition, we identified a novel selective EP4 antagonist HL-43 for promoting chondrocyte differentiation and anabolism with low toxicity and desirable bioavailability. HL-43 enhanced cartilage anabolism, suppressed catabolism, prevented fibrocartilage formation, and reduced joint pain in multiple pre-clinical animal models including the MF surgery-induced CD rat model, the DMM surgery-induced CD mouse model, and an aging-induced CD mouse model. Furthermore, HL-43 promoted chondrocyte differentiation and extracellular matrix (ECM) generation, and inhibited matrix degradation in human articular cartilage explants. At the molecular level, we found that HL-43/EP4 regulated cartilage anabolism through the cAMP/PKA/CREB/Sox9 signaling. Together, our findings demonstrate that EP4 can act as a promising therapeutic target for cartilage regeneration and the novel EP4 antagonist HL-43 has the clinical potential to be used for cartilage repair and regeneration.

## Introduction

Mechanical loading, inflammation, trauma, acute physical injury, and aging are some of the causes for cartilage defects (CDs), and the limited self-healing ability of the articular cartilage leads to production of collagen type I (Col1) and fibrocartilage instead of the stable mature cartilage^1,2^. Lacking the natural functions of the native hyaline cartilage, this fibrocartilage has diminished resiliency, is easily worn and will eventually progress into total joint destructions and osteoarthritis (OA)^3^. Approximately 75% of patients with traumatic injuries develop posttraumatic OA^4,5^. Currently, limited clinical approaches are available to repair defective cartilage, mainly due to the poor regenerative and reparative capacity of cartilage. For example, the widely used microfracture (MF) surgery was shown to enhance migration of stem cells from bone marrow to the injured cartilage^1,6–8^, but only to regenerate fibrocartilage with inferior biochemical and biomechanical characters^1,9^. Moreover, MSCs or stem cells therapy were widely tested in clinical trials in recent years, but these therapies so far do not contribute to improvement in functional outcomes^1,2,10,11^, therefore making cartilage regeneration an urgent unmet clinical need.

Cartilage homeostasis is maintained by the balance between anabolism and catabolism of cartilage matrix. Cartilage anabolism is stimulated by cytokines and growth factors, including bone morphogenetic proteins (BMPs), transforming growth factor (TGF)-β and insulin-like growth factor I (IGF-I), which induce chondrogenesis related genes, such as *Col2a1*, *Acan*, and *Sox9*. These anabolic factors protect the cartilage integrity by enhancing proteoglycan and collagen synthesis, and thereby promoting a healthy extracellular matrix (ECM) structure^12,13^. The catabolic regulators, including interleukin-1 (IL-1), tumor necrosis factor-α (TNF-α), and prostaglandin E2 (PGE2) promote degradation of the articular cartilage ECM through upregulation of matrix metalloproteinases (MMPs) and aggrecanases from the ADAMTS family of proteases^14–16^. Therefore, enhancing cartilage anabolism whilst inhibiting catabolic factors would be a promising strategy for effective cartilage repair and regeneration.

The cartilage of OA patients produces high levels of PGE2, a key pro-inflammatory pain mediator in OA^17^. PGE2 is generated by the initial actions of the cyclooxygenases (COX) on arachidonic acid, which can be further upregulated by IL-1β and TNF-α in OA cartilage^18^. Inhibiting PGE2 production by nonsteroidal anti-inflammatory drugs (NSAIDs), including selective COX-2 inhibitors, has both anti-inflammatory and pain-relieving effects. They are being used for the symptomatic relief of human arthritis including OA^19^. PGE2 has four receptors, prostaglandin E receptor 1–4 (EP1–4), which belong to the G-protein-coupled receptor family^20^. Among the four receptors, EP4 plays a dominant role in cartilage catabolism during OA progression^21–26^. Moreover, the EP4 receptor mediates the PGE2-elicited inflammation and sensitization of sensory neurons^27–30^, leading to the development of targeted therapies for the OA-induced inflammation and pain^31,32^. For example, the EP4 antagonist Grapiprant has been approved for treating OA pain in dogs^33–35^. However, the role of EP4 in cartilage homeostasis still remains elusive as several studies have reported contradictory evidence, using agonists or antagonists exclusively^21–26,31,36,37^. Therefore, further research using *EP4* knockout animal models is warranted to address this discrepancy. Furthermore, the in vivo function of EP4 receptor in articular cartilage regeneration, and its molecular mechanisms remain undetermined.

In this study, we investigated the essential roles of EP4 receptor in articular cartilage regeneration and homeostasis, using two cartilage conditional knockout mice and four cartilage repair models. We developed a novel EP4 antagonist HL-43 that can promote cartilage repair, enhance stable mature articular cartilage formation instead of fibrocartilage formation, delay chondrocyte terminal differentiation through upregulating Sox9 anabolic pathways and inhibit the STAT3 catabolic pathway. These findings provide significant insights into the clinical potential of EP4 antagonist HL-43 in promoting cartilage regeneration and treating CDs.

## Results

### EP4 receptor expression is elevated in human and mouse injured articular cartilage

**Fig. 1:**
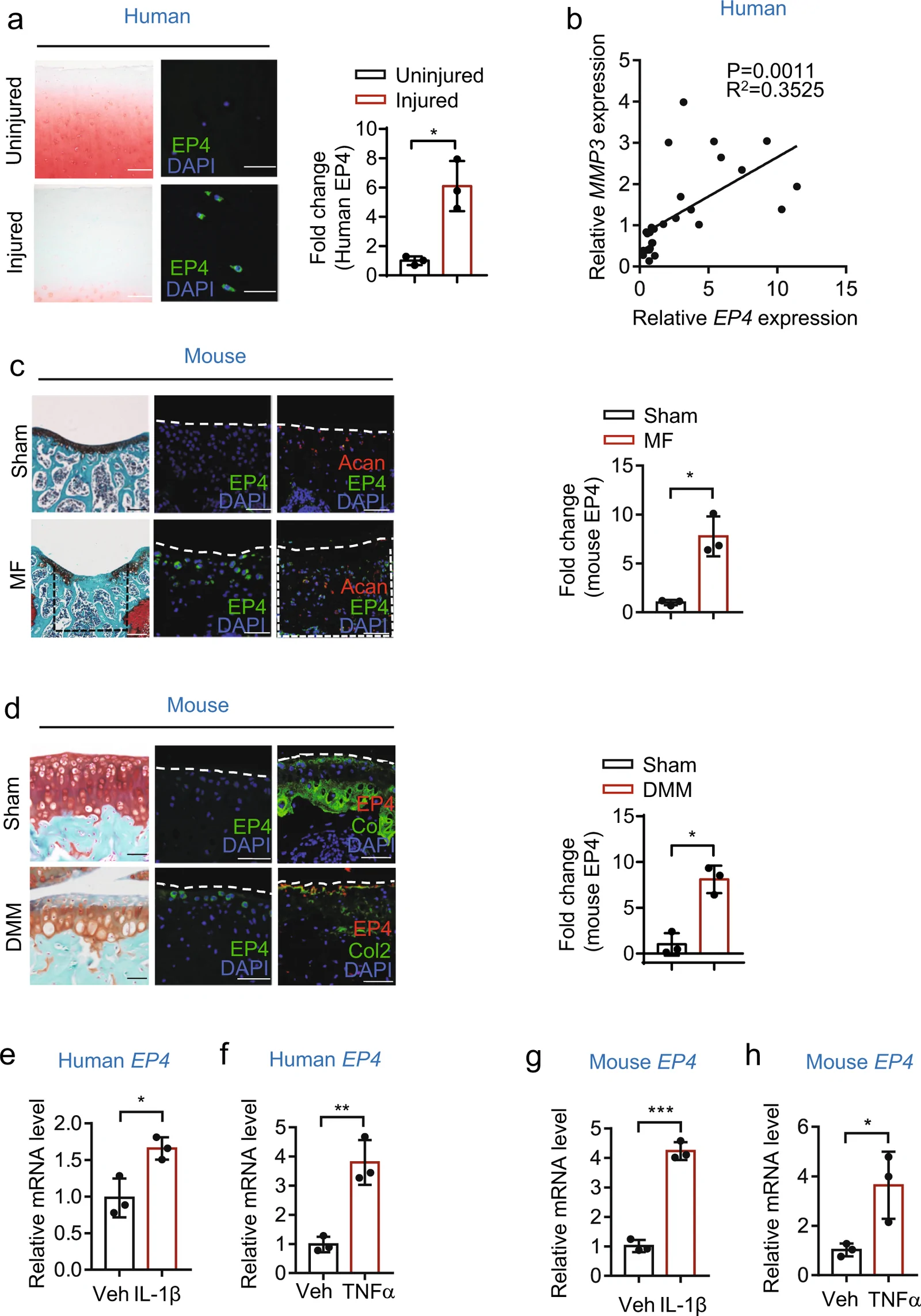
EP4 expression level increased in human and mouse injured articular cartilage. **a** Representative images of safranin O/Fast Green (S.O.) and immunofluorescence staining for EP4 in the uninjured or injured human adult articular cartilage from human OA patients (left). The EP4 expression was quantified (right). Nucleus were shown by DAPI staining. Scale bar, S.O. staining, 200 μm, immunofluorescence staining; 50 μm. Error bars are means ± s.d., *n* = 3, *P < 0.05 by student *t*-test. **b** Scatter plot showing the linear correlation between *MMP3* expression and *EP4* in articular cartilage of human OA patients. Two to three articular cartilage explants from each patient were collected. Samples from ten patients were subject to qRT-PCR. Pearson’s correlation analysis was performed. **c** Representative images of S.O. staining on sham operated or MF at 4 weeks after surgery. Scale bar, 200 μm. EP4 expression level increased in the mouse articular cartilage defect region 4 weeks after MF surgery, co-stained with Acan. Sham-operated mice were used as controls. Scale bar, 50 μm. The EP4 expression was quantified (right). Error bars are means ± s.d., *n* = 3. *P < 0.05 by student *t*-test. White dotted lines indicated the surface of articular cartilage and dotted boxes indicated the defect region. **d** Sham or DMM surgery was carried out at 10-week-old mice. Representative images of S.O. staining of sham-operation or DMM group at 6 weeks after surgery. EP4 expression levels increased in the mouse articular cartilage defect region 6 weeks after the DMM surgery (left). Scale bar, 50 μm. The EP4 expression was quantified, co-stained with Acan (right). Error bars are means ± s.d., *n* = 3. *P < 0.05 by student *t*-test. White dotted lines indicated the surface of articular cartilage. **e**, **f** qRT-PCR analysis for human *EP4* in primary chondrocyte cells from human OA patients’ uninjured articular cartilage treated with or without IL-1β (1 ng/ml) (**e**) or TNF-α (10 ng/ml) (**f**) stimulation for 24 h. Error bars are means ± s.d., *n* = 3. *P < 0.05, **P < 0.01 by student *t*-test. **g**, **h** qRT-PCR analysis for mouse *EP4* in primary mouse articular chondrocyte cells with IL-1β (1 ng/ml) (**g**) or TNF-α (10 ng/ml) (**h**) stimulation for 24 h. Error bars are means ± s.d., *n* = 3. *P < 0.05, ***P < 0.001 by student *t*-test.

To identify potential regulators in cartilage repair, we obtained human cartilage samples from knee OA patients undergoing total knee replacement. The cartilage samples were microdissected from the OA lesion (injured cartilage) and the relative intact non-weight-bearing regions (uninjured cartilage). Safranin-O/Fast-green (S.O.) staining was performed to ensure the initial quality of the different samples. Immunostaining of EP4 showed that the level of EP4 was induced in the injured human articular cartilage relative to uninjured cartilage (Fig. 1a). It has been reported that expression of the catabolic cartilage enzyme MMP3 is elevated in the injured cartilage and barely detectable in uninjured regions^38–40^. In our study, we found that expression of EP4 is positively correlated with MMP3 (*P* = 0.0011, *R*^2^ = 0.3525) in human articular cartilage (*n* = 10) (Fig. 1b), suggesting a critical role for EP4 in cartilage injury. Furthermore, remarkable upregulation of EP4 was observed in the injured cartilage compared to the sham operated cartilage in both microfracture (MF) surgery-induced cartilage defect (CD) mouse model and destabilization of the medial meniscus (DMM) surgery-induced CD mouse model (Fig. 1c, d). Moreover, following treatment with IL-1β or TNF-α, the two cytokines released by injured cartilage^41,42^, EP4 expression levels were markedly elevated in both primary human and mouse articular chondrocytes (Fig. 1e–h). Similar results were confirmed from GEO# GSE104793 databases where a 2.16-fold increase in EP4 expression level was induced by IL-1β (Supplementary Fig. S1).

### Cartilage-specific deletion of EP4 induces articular cartilage regeneration in different CD mouse models

**Fig. 2:**
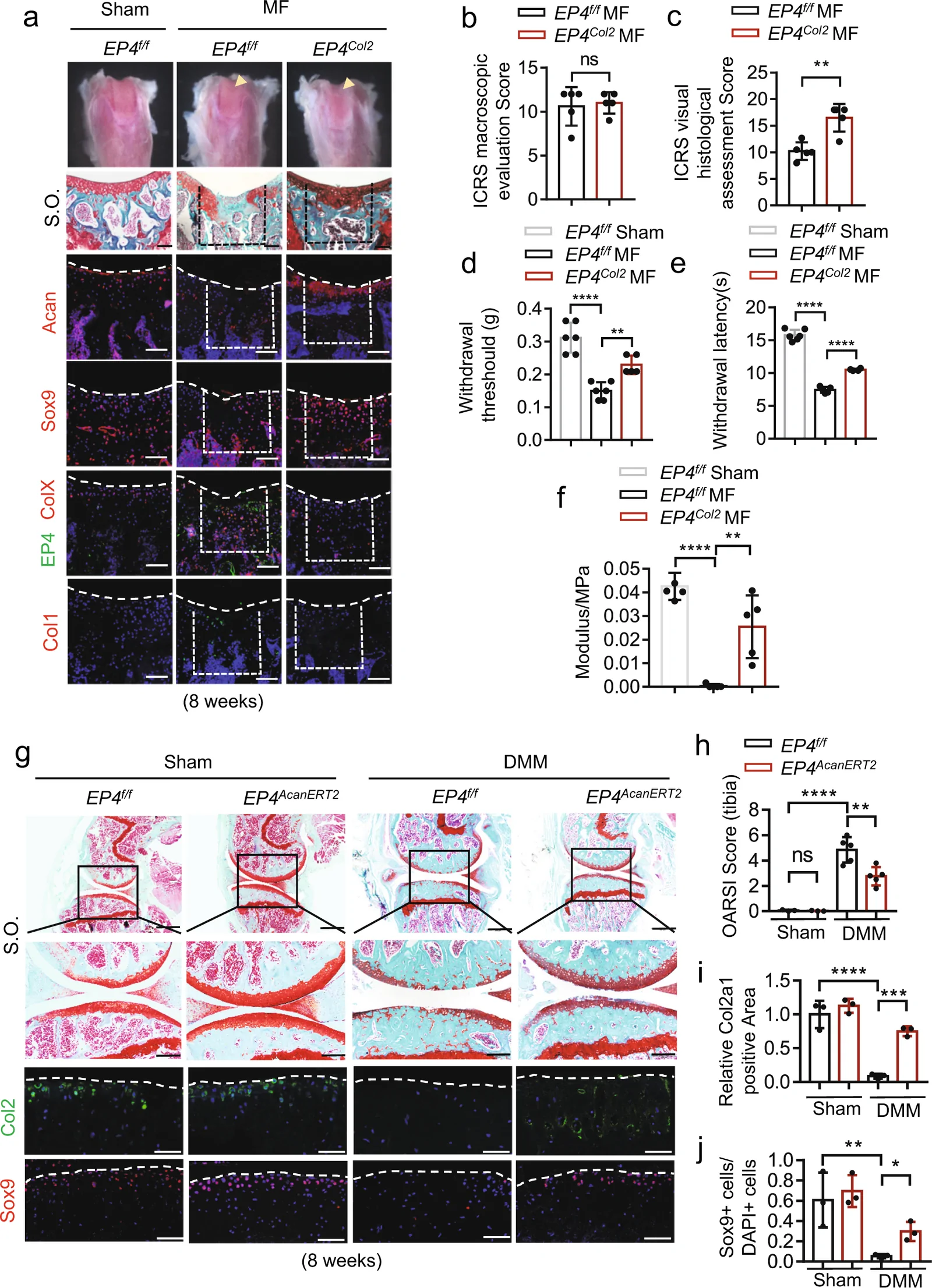
Cartilage-specific deletion of EP4 regenerates the stable mature articular cartilage by promoting cartilage chondrogenesis and anabolism. **a**–**c** Representative images of distal femur of Sham or MF-operation 8 weeks after surgery in *EP4*^*f/f*^ or *Ep4*^*f/f*^; *Col2-Cre* (*Ep4*^*Col2*^) mice (**a**). Yellow arrows show the defect region. International Cartilage Repair Society (ICRS) macroscopic evaluation score assessment was performed (**b**). The S.O. staining of the articular cartilage sections. Scale bar, 200 μm. ICRS Visual Histological Score of regenerated tissues were performed (**c**). The articular cartilage sections from sham or MF surgery were stained with indicated antibodies (**a**). Nuclei are shown by DAPI staining. Scale bars, 200 μm. Dotted lines indicated the surface of articular cartilage and dotted boxes indicated the defect region. *n* = 5 for each group. Error bars are means ± s.d., **P < 0.01 by student *t*-test. ns, not significant; IF, immunofluorescence. **d**, **e** The pain was measured by Von Frey assay (**d**) and radiant heat paw-withdrawal test (**e**). Error bars are means ± s.d., *n* ≥ 5 for each group, **P < 0.01, ***P < 0.001, ****P < 0.0001 by one-way ANOVA with multiple comparison using Tukey method. **f** Quantification for the modulus of the regenerate 8 weeks after MF surgery. *n* = 5 for each group, error bars are means ± s.d., **P < 0.01, ****P < 0.0001 by one-way ANOVA with multiple comparison using Tukey method. **g**–**j** Representative images of S.O. staining of Sham-operation or DMM at 8 weeks after surgery in *EP4*^*f/f*^ or *Ep4*^*f/f*^; *Aggrecan-CreERT2* (*Ep4*^*AcanERT2*^) mice. Scale bar (top), 500 μm; scale bar (bottom), 200 μm. The corresponding OARSI scores were performed (**h**). Error bars are means ± s.d., *n* = 5 for each group, **P < 0.01, ***P < 0.001, ****P < 0.0001 by ordinary one-way ANOVA with multiple comparison using Tukey method. ns not significant. Knee joint sections were stained with antibodies against Col2a1 (Scale bars are 100 μm) and Sox9 (Scale bars are 50 μm) (**g**). Nucleus were shown by DAPI staining. Dotted lines indicated the surface of articular cartilage. The Col2a1 and Sox9 expression were quantified in **i** and **j**, respectively. Error bars are means ± s.d., *n* = 3 for each group, *P < 0.05, **P < 0.01, ***P < 0.001, ****P < 0.0001 by ordinary one-way ANOVA with multiple comparison using Tukey method.

We next generated cartilage-specific *EP4* knockout mice to assess if EP4 regulates the cartilage regeneration. Firstly, using the MF surgery-induced CD mouse model, we found that cartilage-specific deletion of *EP4*, *Col2-Cre; EP4*^*f/f*^ (*EP4*^*Col2*^), accelerated cartilage repair mildly at 2 weeks (Supplementary Fig. S2a–e), but significantly at 4 weeks and 8 weeks after MF surgery (Supplementary Fig. S2f–h and Fig. 2a–c). Moreover, the expression of chondrogenic differentiation and cartilage anabolic factors Col2, Acan, and Sox9 largely increased, while the catabolic factor Mmp13 and hypertrophic factor ColX decreased in *EP4*^*Col2*^ mouse cartilage compared with the control mice (Fig. 2a and Supplementary Fig. S2f). The expression of EP4 was also significantly higher in the MF surgery group. In humans and mice, the regenerated cartilage after the MF surgery is mainly fibrocartilage, which is positive for Col1 and Mmp13 and negative for Col2^2^. Of note, *EP4* deletion inhibited the expression of Col1 and Mmp13, while increasing the expression of Col2, Acan and Sox9 (Supplementary Fig. S2f). As a result, the modulus of the regenerate assessed by atomic force microscopy was significantly higher in the *EP4*^*Col2*^ group compared to the *EP4*^*f/f*^ group after MF surgery (Fig. 2f). Furthermore, *EP4*-deficiency showed a higher paw withdrawal threshold in von Frey assay compared to the control group at both 4 weeks and 8 weeks of post MF surgery (Fig. 2d and Supplementary Fig. S2i). The thermal withdrawal latency in the thermal hyperalgesia test was also significantly higher at these time-points post MF surgery (Fig. 2e and Supplementary Fig. S2j). All the results indicate that knocking out *EP4* in cartilage enhances stable mature articular cartilage formation instead of fibrocartilage formation, and reduces joint pain.

This observation was further validated in our separate DMM surgery-induced CD mouse model. To avoid the chondrogenesis defect during development, we employed the inducible cartilage-specific transgenic CRE mouse line (*Aggrecan-creERT2*) to delete EP4 in cartilage postnatally (*EP4*^*AcanERT2*^). The cartilage injury and glycosaminoglycans loss can be detected 8 weeks after DMM surgery (Fig. 2g), while ablation of *EP4* notably rescued articular cartilage destruction and the proteoglycan content compared to the DMM-operated *EP4*^*f/f*^ control group (Fig. 2g). The Osteoarthritis Research Society International (OARSI) scores were significantly decreased in the *EP4*^*AcanERT2*^ mice compared to *EP4*^*f/f*^ controls, after DMM surgery (Fig. 2h). Similar to the MF surgery-induced CD mouse model, the expression of chondrogenic differentiation and cartilage anabolic factors Col2 and Sox9 were significantly increased (Fig. 2g, i, j). We also observed high expression of EP4 in the *EP4*^*f/f*^ controls after DMM surgery, and the catabolic factor Mmp13 and hypertrophic marker ColX notably decreased in *EP4*^*AcanERT2*^ articular cartilage compared to the *EP4*^*f/f*^ control group (Supplementary Fig. S3a–c). Collectively, these results indicate that genetic deletion of *EP4* in cartilage promotes articular cartilage repair by enhancing cartilage anabolism and inhibiting cartilage catabolism.

Our data also showed that synovitis was reduced in the *EP4*^*AcanERT2*^ mice compared to *EP4*^*f/f*^ mice after DMM surgery, with decreased synovitis scores and reduced synovial hyperplasia (Supplementary Fig. S3d, e). Moreover, ablation of *EP4* showed a higher paw withdrawal threshold in von Frey assay in comparison with the control group (Supplementary Fig. S3f), a significantly increased thermal withdrawal latency in a thermal hyperalgesia test (Supplementary Fig. S3g), and a decreased weight-bearing load in the *EP4*^*AcanERT2*^ group compared with *EP4*^*f/f*^ group, 8 weeks after DMM surgery (Supplementary Fig. S3h). All these results demonstrate that cartilage-specific deletion of *EP4* mediates reductions in DMM surgery-induced inflammation and pain.

### *EP4*-deficiency enhances chondrogenesis and inhibits chondrocyte hypertrophy and catabolism

**Fig. 3:**
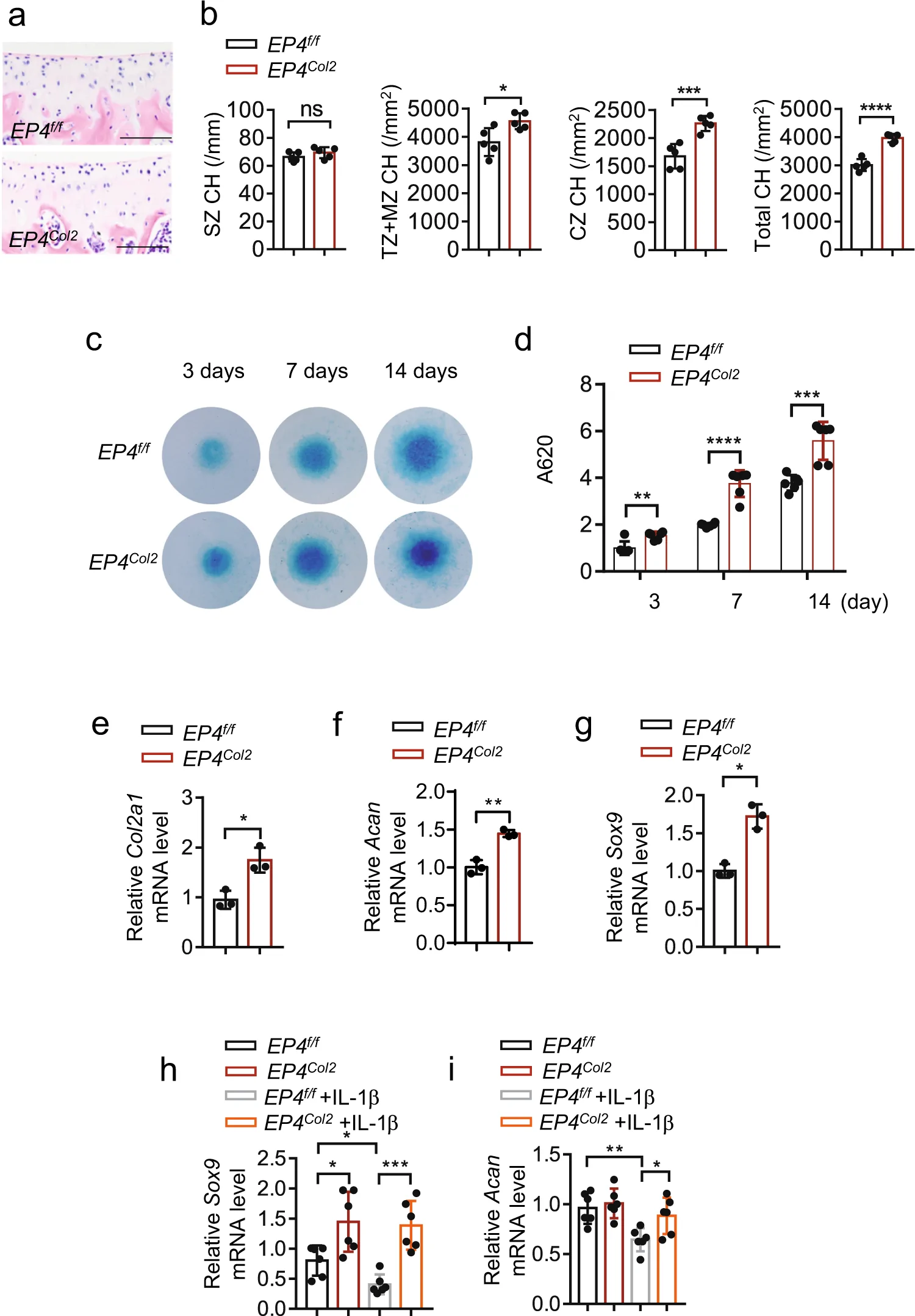
EP4 regulates chondrocyte differentiation and chondrocyte anabolism. **a**, **b** H&E staining of femoral articular cartilage from *EP4*^*f/f*^ and *Ep4*^*Col2*^ mice at 3 months of age (**a**). Scale bar, 50 μm. Chondrocyte numbers (CH) were calculated in superficial zone (SZ), transition, and middle zones (TZ + MZ), calcified zone (CZ), and entire femoral articular cartilage quantified (Total CH) (**b**). *n* = 5 mice per group. **c**, **d** Primary articular chondrocytes were isolated from *Ep4*^*Col2*^ knee joints of new born pups. Micromass of the chondrocytes cultured in the chondrogenic media for 3, 7, and 14 days were stained with Alcian blue (**c**). Alcian blue-stained cultures were quantified (**d**). Error bars are means ± s.d., *n* = 6. **P < 0.01, ***P < 0.001, ****P < 0.0001 by by two-way ANOVA with multiple comparison using Sidak method. **e**–**g** qRT-PCR analyses of *Acan, Col2a1*, and *Sox9* expression in primary articular chondrocytes of *EP4*^*f/f*^ and *Ep4*^*Col2*^ and 7 days after chondrogenic differentiation. Error bars are means ± s.d., *P < 0.05, **P < 0.01 by student *t*-test. **h**, **i** qRT-PCR analyses of *Acan* and *Sox9* expression in primary articular chondrocytes from *EP4*^*f/f*^ and *Ep4*^*Col2*^mice treated with IL-1β for 24 h. Error bars are means ± s.d., *n* = 6, *P < 0.05, **P < 0.01, ***P < 0.001 by one-way ANOVA with multiple comparison using Tukey method.

To determine the role of EP4 receptor in regulating chondrogenesis, we calculated the number of chondrocytes in each zones of the knee joints from *EP4*^*f/f*^ and *EP4*^*Col2*^ mice at 3 months of age. The *EP4*^*Col2*^ mice exhibited an increased total number of chondrocytes, with the transition and middle zones, as well as the calcified zone all showing a significant increase (Fig. 3a, b). Next, we isolated the primary articular chondrocytes from *EP4*^*f/f*^ and *EP4*^*Col2*^ mice and firstly examined the cell growth by SulforhodamineB (SRB) assay. Our data showed that *EP4* knockout mildly induced the growth of chondrocytes at 4 days, and later (Supplementary Fig. S4a). Similar results were obtained from the in vivo Ki67 staining of knee joints (Supplementary Fig. S4b), suggesting that EP4 regulated articular chondrocyte proliferation to a small extent. Next, we examined the chondrocyte differentiation in the primary articular chondrocytes, which were treated with chondrogenic medium for 3, 7, and 14 days. The results showed that deficiency of *EP4* strongly induced differentiation in *EP4*^*Col2*^ chondrocytes (Fig. 3c, d). Consistently, after 7 days of chondrogenic induction, the chondrogenic markers including *Col2a1*, *Acan*, *Sox9* significantly increased in *EP4*^*Col2*^ cells, while the catabolic factors *Mmp3* and *Mmp13*, and the hypertrophic marker *ColX* and *Runx2* were lower compared to the *EP4*^*f/f*^ cells (Fig. 3e–g and Supplementary Fig. S4c). Similar results were obtained in a pro-inflammatory condition. IL-1β downregulated anabolic factors *Sox9*, *Acan* and upregulated catabolism marker *Mmp3/13*, while deletion of *EP4* was protective against these changes (Fig. 3h, i and Supplementary Fig. S4d). To confirm the effects observed in this study are not due to the indirect effects of *EP4* expression on stem cells, we differentiated bone marrow mesenchymal stem cells (BMSCs) using chondrogenic media. We observed that knocking out *EP4* in BMSCs inhibited chondrogenic differentiation as measured by alcian blue staining (Supplementary Fig. S4e). Moreover, *EP4* knockout stem cells did not induce expression of cartilage differentiation markers Col2a1, Acan, Sox9 expression during chondrogenic differentiation (Supplementary Fig. S4f). Taken together, these results suggest that deletion of *EP4* promotes chondrogenesis and inhibits chondrocyte hypertrophy and catabolism under both physiological and pro-inflammatory conditions.

### Identification of a novel EP4 antagonist HL-43 that promotes chondrocyte differentiation and anabolism

**Fig. 4:**
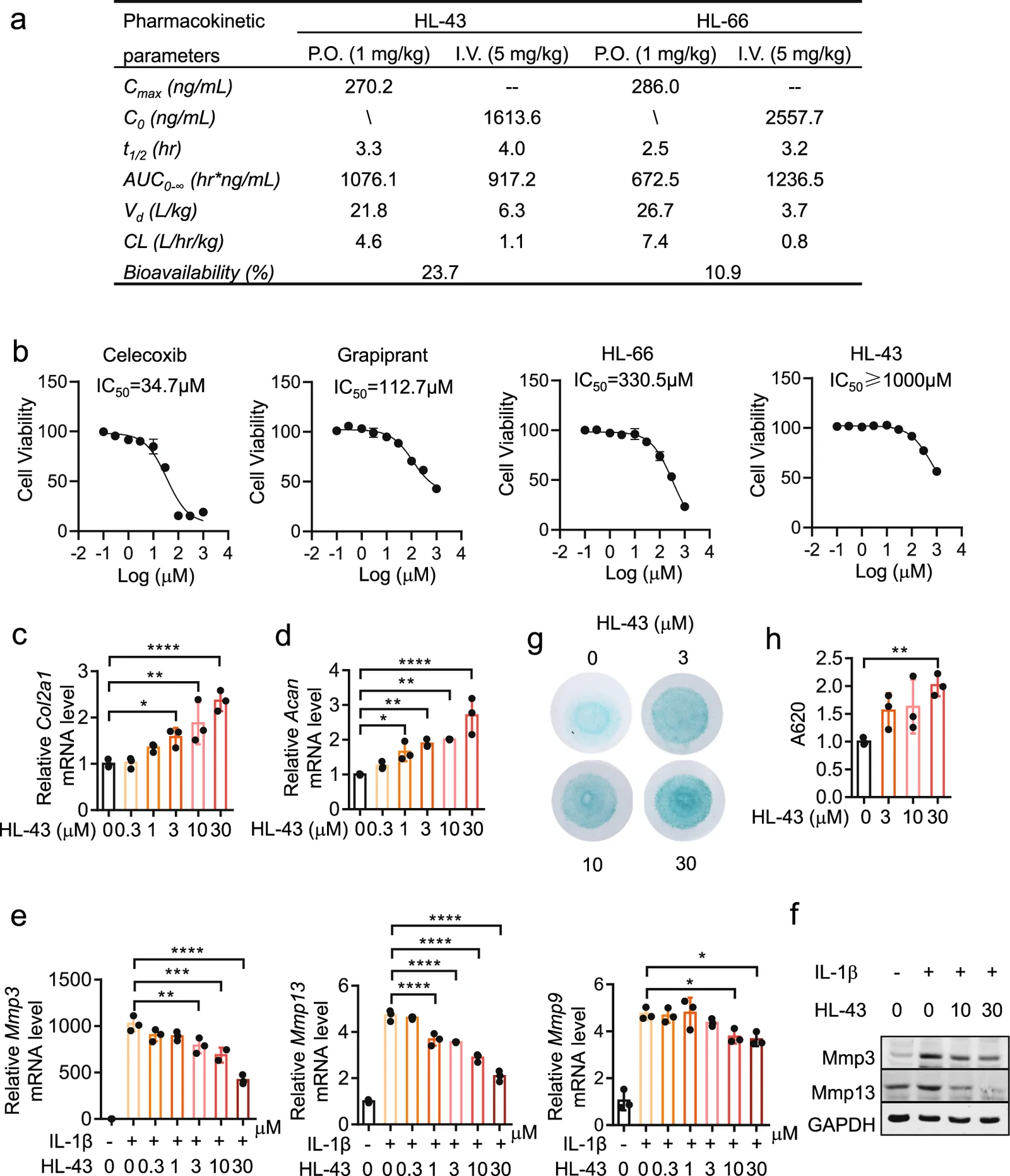
Identification of a novel EP4 antagonist HL-43 for promoting chondrocyte differentiation and anabolism with low toxicity and desirable bioavailability. **a** Pharmacokinetic parameters of HL-66 and HL-43 after oral (P.O.) administration or intravenous (I.V.) injection in CD1 mice. **b** Primary articular chondrocytes were treated with Celecoxib, Grapiprant, HL-66, and HL-43 for 72 h. Cell viability was assessed using CCK-8 assay and IC_50_ values were calculated respectively. Error bars are means ± s.d., *n* = 3. **c**, **d** qRT-PCR analysis of anabolic factors *Col2a1* (**c**), *Acan* (**d**) expressed in primary articular chondrocytes treated with indicated concentrations of HL-43 for 24 h. Error bars are means ± s.d., *n* = 3. *P < 0.05, **P < 0.01, ***P < 0.001, ****P < 0.0001 by one-way ANOVA with multiple comparison using Tukey method. **e** qRT-PCR analysis of catabolic factors *Mmp3, Mmp13*, *Mmp9* expressed in primary articular chondrocytes treated with 1 ng/ml IL-1β and indicated concentrations of HL-43 for 24 h. Error bars are means ± s.d., *n* = 3. *P < 0.05, **P < 0.01, ***P < 0.001, ****P < 0.0001 by one-way ANOVA with multiple comparison using Tukey method. **f** Western blotting analysis of catabolic factors Mmp3 and Mmp13 expressed in primary articular chondrocytes treated with 1 ng/ml IL-1β and indicated concentrations of HL-43 for 24 h. **g**, **h** HL-43 induced chondrocyte differentiation in a dose-dependent manner. Micromass of the chondrocytes cultured in the chondrogenic media for 7 days and stained with Alcian blue (**e**). Alcian blue-stained cultures were quantified (**f**). Error bars are means ± s.d., *n* = 3. *P < 0.05, **P < 0.01, ***P < 0.001, ****P < 0.0001 by one-way ANOVA with multiple comparison using Tukey method.

To identify potent EP4 antagonists for cartilage regeneration, we screened the class of 1H-1,2,3-triazole-based small molecule compounds, all of which specifically target the EP4 receptor^43^. Using the cartilage anabolism marker Col2a1 and catabolism marker Mmp3 as a readout, we found that IL-1β treatment reduced the expression of *Col2a1* and induced the expression of *Mmp3* in primary murine chondrocytes. Two EP4 antagonists HL-43 and HL-66 exhibited the highest potency in inducing *Col2a1* expression and reducing *Mmp3* expression in presence of IL-1β (Supplementary Fig. S5a–c). Interestingly, both HL-43 and HL-66 have better efficacies compared to two FDA approved NSAIDs — Celecoxib, a COX-2 inhibitor, and the EP4 antagonist Grapiprant (used for dog OA pain). Next, the pharmacokinetic properties of HL-43 and HL-66 were evaluated in CD1 mice (Fig. 4a). Our data showed that HL-43 had a better half-life (*t*_1/2_), clearance rate (CL), and favorable oral bioavailability than HL-66. The toxicity assay also determined the IC_50_ of HL-43 in chondrocytes is greater than 1000 μM, which is much better than Celecoxib (IC_50_ = 34.7 μM), Grapiprant (IC_50_ = 112.7 μM), and HL-66 (IC_50_ = 330.5 μM) (Fig. 4b). Therefore, we chose the compound HL-43 with the best physicochemical properties for further studies.

As the FDA approved Celecoxib has been reported to have fetotoxicity, we examined the embryo toxicity of HL-43 using a standard zebrafish embryo toxicity assay. Our results showed that in comparison to Celecoxib and Grapiprant, HL-43 showed the lowest mortality rate at 24 hpf, the lowest deformities rate, and the highest hatching rate at 72 hpf (Supplementary Fig. S5d).

Furthermore, HL-43 promoted the expression of anabolic factors (*Col2a1* and *Acan*) in primary mouse chondrocytes, and attenuated the expression of catabolic factors (*Mmp3*, *Mmp9*, and *Mmp13*) in a dose-dependent manner (Fig. 4c–e). Western blotting analysis also showed that HL-43 downregulated Mmp3/13 expression in a dose-dependent manner (Fig. 4f). Similar to the *EP4* knockout results, HL-43 also promoted the chondrogenesis of the primary chondrocytes in a dose-dependent manner (Fig. 4g, h). Taken together, the results indicate that the EP4 antagonist HL-43 is a promising lead compound for promoting chondrogenesis and blocking catabolism, with low toxicity and desirable bioavailability.

### EP4 antagonist HL-43 promotes chondrocyte differentiation and ECM generation, and inhibits matrix degradation in both human and mouse articular cartilage explants

**Fig. 5:**
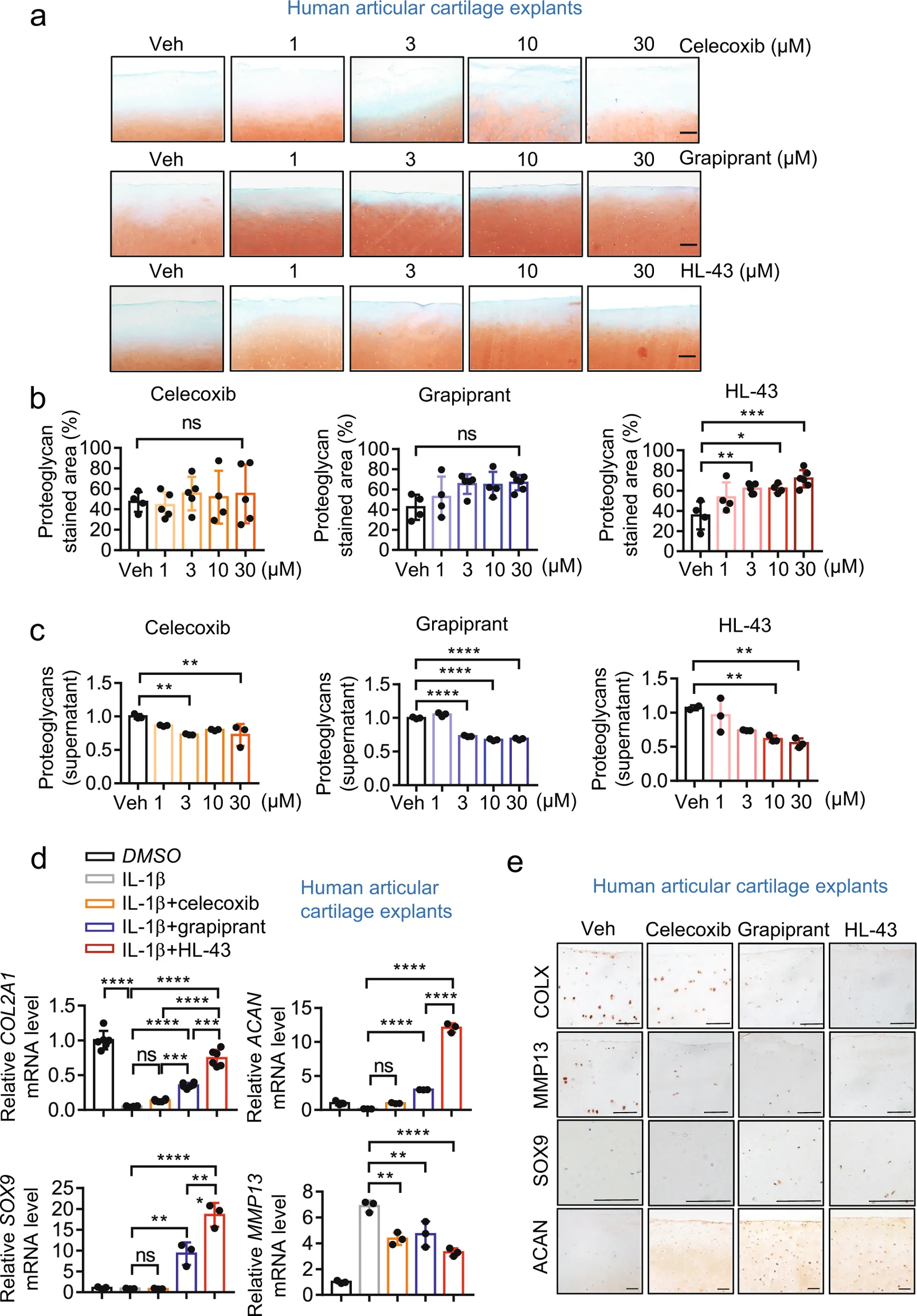
EP4 antagonists prevented cartilage ECM breakdown by promoting ECM generation and inhibiting cartilage catabolism in human articular cartilage. **a** HL-43 prevented cartilage ECM degradation. The relative intact non-weight-bearing explants of human cartilage were isolated from knee OA patients undergoing total knee replacement. S.O. staining of OA patient cartilage explants after 7 days of treatment with Celecoixb, Grapiprant, and HL-43 at indicated dosage. Scale bars, 200 μm. Veh, vehicle. **b** Proteoglycan staining area (%) was measured by S.O. staining. Error bars are means ± s.d., *n* ≥ 4 for each group. **c** Release of total glycosaminoglycan (GAG) into the media of human articular cartilage explant was measured by 1,9-dimethylmethylene blue (DMMB) assays. Human articular cartilage explants were treated with the indicated chemical compounds for 7 days. The secreted GAG was normalized to the wet weight of the cartilage explant to calculate the release of GAG, then normalized to vehicle. Error bars are means ± s.d., *n* = 3 for each group. **d** qRT-PCR analysis of anabolic factors *COL2A1*, *ACAN*, *SOX9*, and *MMP13* expressed in the human articular cartilage explants treated with 1 ng/ml IL-1β and indicated chemical compound for 24 h (Celecoxib 10 μM, Grapiprant and HL-43 30 μM). **e** Representative images of immunohistochemistry staining of indicated antibodies in vehicle, Celecoxib, Grapiprant, HL-43 treated human cartilage explants, scale bars, 100 μm. *P < 0.05, **P < 0.01, ***P < 0.001, ****P < 0.0001 by one-way ANOVA with multiple comparison using Tukey method. ns, not significant; Veh, vehicle.

Next, we examined whether HL-43 promoted ECM generation in cartilage, and inhibited matrix degradation ex vivo. The relative intact non-weight bearing explants of human OA cartilage were cultured in the presence of Celecoxib, Grapiprant or HL-43 for 1 week, and cartilage properties were monitored. Our data showed that the percentage area of proteoglycan determined by S.O. staining was increased with HL-43 treatment in a dose-dependent manner, but not with Celecoxib or Grapiprant (Fig. 5a, b). Similarly, all the compound treated groups showed significant dose-dependent inhibitory effect on glycosaminoglycans (GAGs) release, examined using the dimethylmethylene blue (DMMB) assay (Fig. 5c). Furthermore, among the three compounds, HL-43 had the best efficacy on promoting the expression of chondrocyte anabolic factors (*COL2A1, ACAN*, and *SOX9*) and suppressing the catabolic factor (*MMP13*) in the human articular cartilage explants, under IL-1β treatment (Fig. 5d). The immunohistochemistry staining also confirmed that HL-43 inducted anabolic factors (ACAN and SOX9), and suppressed catabolic factor (MMP13), and hypertrophic marker (COLX) (Fig. 5e). Similar results were observed in mouse femoral head cartilage explants where HL-43 had a favorable effect on the proteoglycan content, as determined by S.O. staining (Supplementary Fig. S6a). This was accompanied by reduced immunohistochemistry staining for the catabolic marker Mmp13 (Supplementary Fig. S6a), and GAG assessed by the DMMB assay (Supplementary Fig. S6b).

### EP4 antagonist HL-43 induces cartilage repair and regeneration in three different CD animal models

**Fig. 6:**
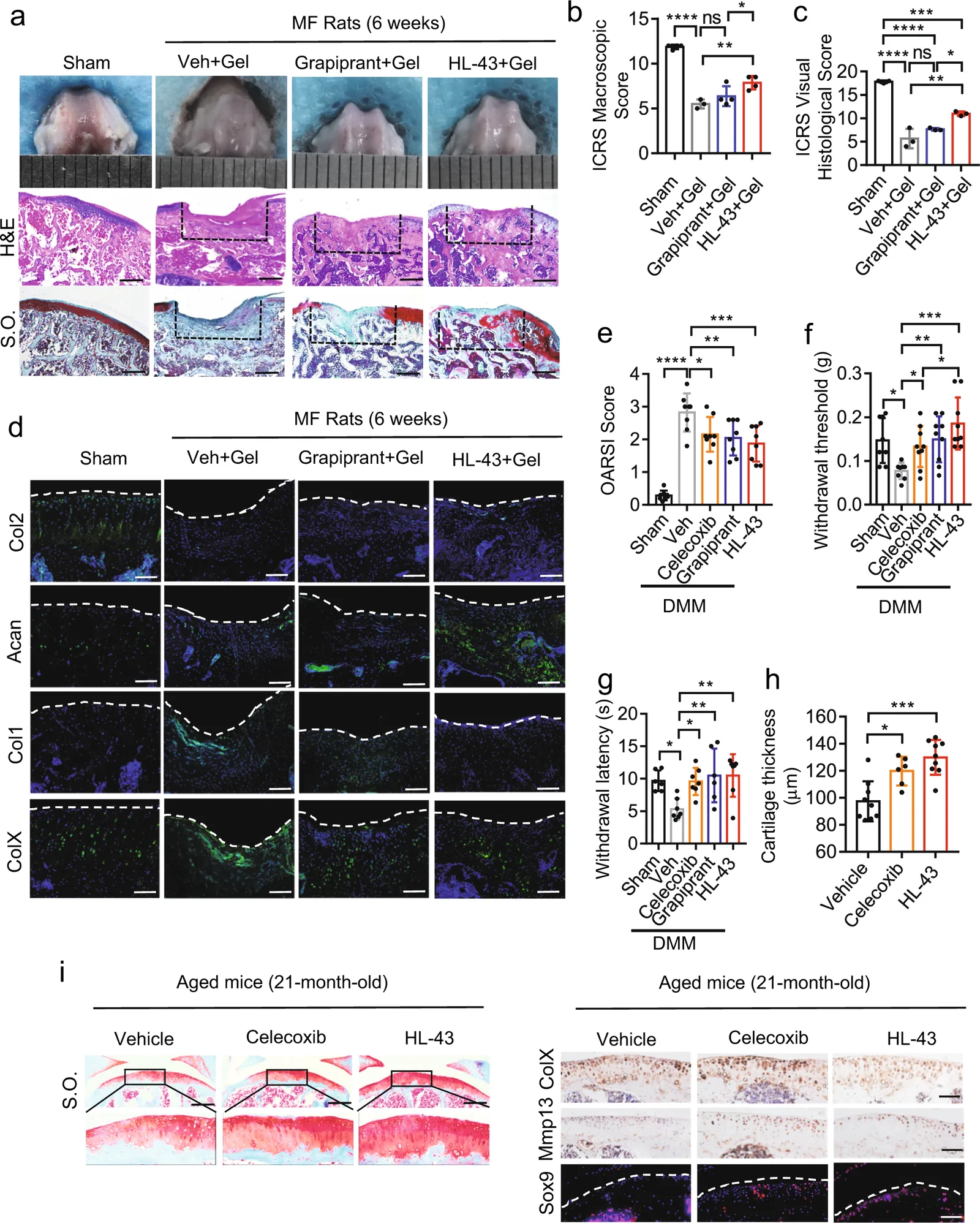
EP4 antagonist HL-43 enhanced cartilage repair and regeneration in three different CD animal models. **a**–**c** EP4 antagonist HL-43 induced cartilage regeneration in MF surgery-induced CD rat model. Macroscopic appearance of the articular cartilage, H&E staining and S.O. staining of the sham and defect cartilage 6 weeks after MF surgery were shown (**a**). Black dotted boxes indicate the defect region. The International Cartilage Repair Society (ICRS) macroscopic score and ICRS Visual Histological Score of regenerated tissues analysis was analyzed (**b**, **c**). *n* = 4 for each group, scale bar = 500 μm. Error bars are means ± s.d., *P < 0.05, **P < 0.01 by ANOVA and LSD post hoc test. ns, not significant. **d** EP4 antagonists induced the mature cartilage formation in MF surgery-induced CD rat model. Joint sections from Sham, Vehicle (Veh), Grapiprant and HL-43-treated rats were stained with antibodies against Col2, Acan, Col1, and ColX. Nucleus were shown by DAPI staining. Scale bars are 100 μm. Dotted lines indicated the surface of articular cartilage. **e** EP4 antagonist HL-43 alleviated OA in the DMM surgery-induced CD mouse model. The corresponding Osteoarthritis Research Society International (OARSI) scores were compared. Error bars are means ± s.d., *n* = 8 for each group, **P < 0.01, ***P < 0.001, ****P < 0.0001 by ordinary one-way ANOVA with multiple comparison using Tukey method. ns, not significant. **f**, **g** The relief of the pain by treatment of HL-43 in the DMM surgery-induced CD mouse model. Von Frey assay (**f**) and radiant heat paw-withdrawal test (**g**) were measured in each group 6 weeks after DMM surgery. *n* = 8 for each group in Von Frey assay, *n* ≥ 6 for radiant heat paw-withdrawal test, *P < 0.05, **P < 0.01, ***P < 0.001 by ANOVA and LSD post hoc test. **h** EP4 antagonist HL-43 induced cartilage regeneration in the aging-induced CD mouse model. Articular cartilage thickness of the tibia plateau at same position was measured. Error bars are means ± s.d., *n* = 9 for the vehicle and HL-43-treated groups, *n* = 6 for Celecoxib treated group, *P < 0.05, ***P < 0.001 by one-way ANOVA with multiple comparison using Tukey method. **i** EP4 antagonist HL-43 induced cartilage regeneration in the aging-induced CD mouse model. Representative images of S.O. staining of knee joint sections in aged mice (21-month-old), which treated with 30 mg/kg vehicle, Celecoxib, and HL-43 daily from 19 to 21 months by gavage. Scale bar, 200 μm. Immunostaining of knee joint sections from each group with indicated antibodies. Scale bar, 100 μm. Dotted lines indicated the surface of articular cartilage.

To determine whether EP4 antagonist HL-43 enhanced articular cartilage regeneration in vivo, three different CD animal models including MF surgery-induced CD rat model, the DMM surgery-induced CD mouse model, and an aging-induced CD mouse model were employed. Firstly, we used MF surgery-induced CD rat model to test if a hydrogel containing Grapiprant or HL-43 could induce cartilage regeneration. We found that only HL-43 started to show mild effects on cartilage repair at 4 weeks after MF surgery (but not at 2 weeks), based on ICRS Macroscopic and ICRS Visual Histological scores (Supplementary Fig. S7a–f). Nevertheless at 6 weeks of post-surgery, the surfaces were smooth and cartilage layers were regenerated in the HL-43 treated group, while the CDs in the vehicle and Grapiprant treated groups were still visible (Fig. 6a–c). Moreover, the immunofluorescence staining showed increased expression of mature articular cartilage markers (Col2 and Acan) and reduced fibrocartilage markers (Col1 and ColX) in the regenerated cartilage (Fig. 6d). These results demonstrate that EP4 antagonist HL-43 can strongly promote cartilage regeneration.

Secondly, we explored whether HL-43 induced articular cartilage repair in a DMM surgery-induced CD mouse model. We treated the mice with HL-43, Celecoxib, or Grapiprant daily by oral gavage after the DMM surgery. The results confirmed that oral treatment with all three compounds rescued articular CDs, the proteoglycan content, OARSI scores, and alleviated the DMM-induced pain 6 weeks after surgery (Fig. 6e–g and Supplementary Fig. S8a). Importantly, the cartilage repair effects in the 10 mg/kg HL-43 treated group were similar to the 30 mg/kg Celecoxib or 30 mg/kg Grapiprant treated groups (Fig. 6e and Supplementary Fig. S8a). The expression of chondrogenic differentiation and cartilage anabolic factors Col2, Acan, and Sox9 were increased, while the catabolic factors Mmp13 and hypertrophic marker ColX were decreased in the EP4 antagonist (Grapiprant and HL-43) treated groups compared to the vehicle control group (Supplementary Fig. S8a). Our data also showed that the synovitis was reduced in all three compound treatment groups, with reduced synovial hyperplasia and decreased anterior synovitis scores (Supplementary Fig. S8a, b).

Lastly, we evaluated the cartilage regeneration in the aging-induced CD mouse model (19-month-old C57/BL6 mice) with oral treatments of vehicle, Celecoxib, and EP4 antagonist HL-43 for 3 months. Our results showed loss of cartilage integrity at 22 months of age with decreased proteoglycan content, thinned cartilage, and disturbed cellularity in the vehicle control group (Fig. 6i), while HL-43 treatment notably preserved cartilage integrity and had thicker articular cartilage as shown using S.O. staining analysis (Fig. 6h, i). Similarly, higher expression of chondrogenic differentiation and cartilage anabolic factor Sox9, and lower expression of catabolic factors Mmp13 and hypertrophic marker ColX were observed in the HL-43 treatment group (Fig. 6i).

Taken together, these results demonstrate that inhibiting the EP4 receptor by HL-43 could effectively enhance articular cartilage regeneration in trauma, DMM, and aging-induced CD conditions by promoting chondrogenic differentiation and cartilage anabolism, and suppressing cartilage catabolism and hypertrophy.

### EP4 antagonist HL-43 enhances chondrogenic differentiation and cartilage anabolism through regulation of the cAMP/PKA/CREB/Sox9 signaling

**Fig. 7:**
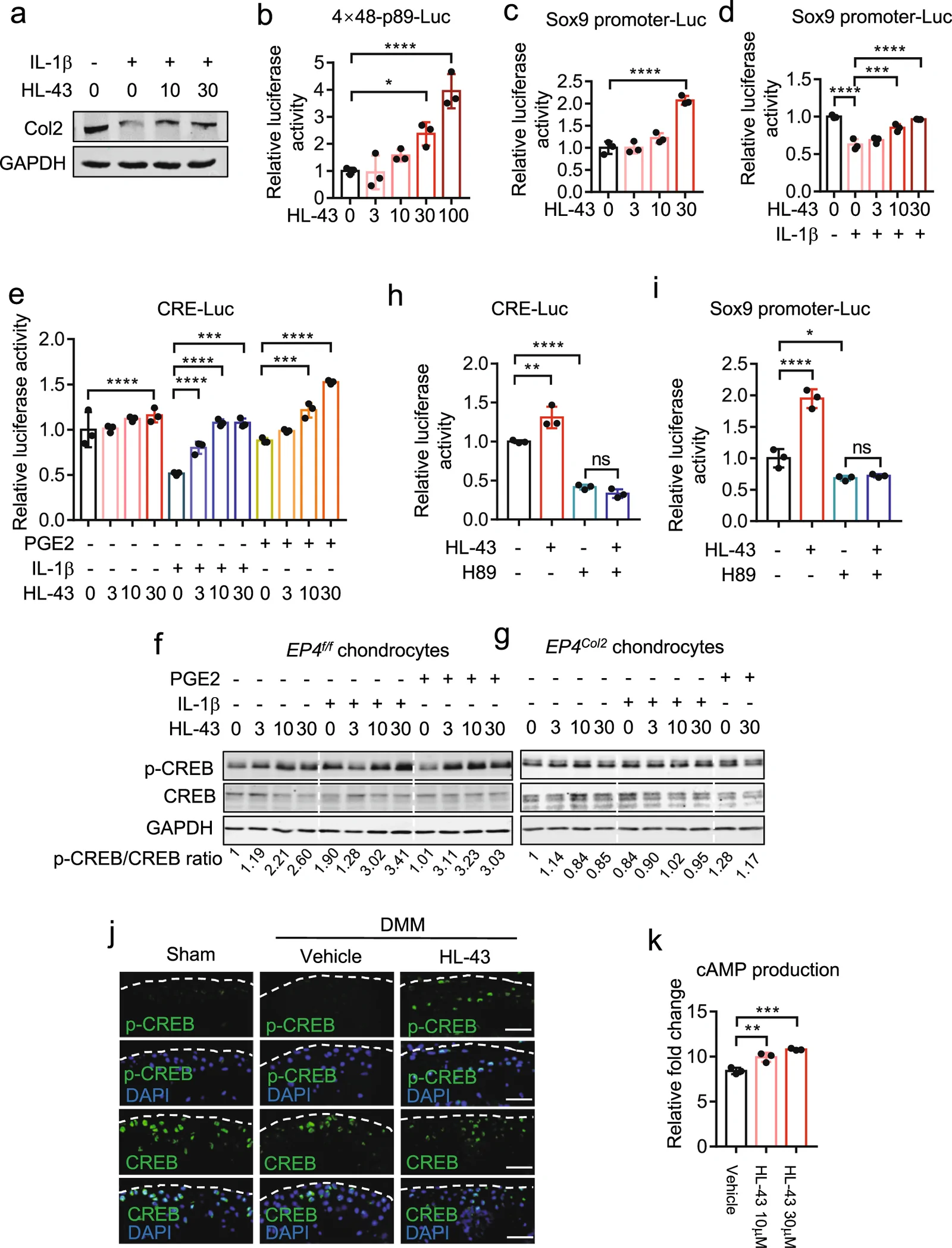
EP4 mediated cartilage anabolism through regulation of the Sox9 expression by cAMP/PKA/CREB signaling. **a** Primary culture of articular chondrocytes was treated with or without 1 ng/ml IL-1β, and the indicated concentrations (μM) of HL-43 for 24 h. Col2a1 and GAPDH protein levels were detected by Western blotting. **b** Activation of the 4 × 48-p89-Luciferase reporter by HL-43 in a dose-dependent manner in ATDC5 cells, *n* = 3. **c**, **d** HL-43 increased the Sox9 transcriptional activity. After transfected with the Sox9 promoter-Luc reporter, ATDC5 cells treated with (**d**) or without (**c**) 2 ng/ml IL-1β and indicated concentrations (μM) of HL-43 for 6 h. The cell lysates were subjected to a Dual-Luciferase assay, *n* = 3. **e** HL-43 activated the CRE-Luc reporter. ATDC cells were transfected with the CRE-Luc reporter and treated with or without 2 ng/ml IL-1β, 100 nM PGE2, and indicated concentrations (μM) of HL-43 for 6 h, the cell lysates were subjected to a Dual-Luciferase assay, *n* = 3. **f**, **g** HL-43 increased the phosphorylation of CREB through EP4. Chondrocytes isolated from new born *EP4*^*f/f*^ mice (**f**) or *EP4*^*Col2*^ mice (**g**) were treated with 2 ng/ml IL-1β, 100 nM PGE2, and the indicated concentration (μM) of HL-43 for 1 h. p-CREB, CREB, and GAPDH protein levels were detected by Western blotting. Protein bands in the blot were quantified by densitometry and analyzed using Image J. The numbers indicate grayscale ratios of p-CREB/CREB. **h** ATDC5 cells were transfected with the CRE-Luc reporter gene. After treated with the 2 ng/ml ml IL-1β, 10 μM H89, and 30 μM HL-43 for 6 h, the cell lysates were subjected to a Dual-Luciferase assay (*n* = 3). **i** HL-43 regulated Sox9 promoter through PKA activity. After transfected with Sox9 promoter-Luc reporter, ATDC5 cells were treated with or without 10 μM H89 or 30 μM HL-43 for 6 h. The cell lysates were subjected to a Dual-Luciferase assay, *n* = 3. **j** HL-43 increased the phosphorylation of CREB in the DMM surgery-induced CD mouse model (refer to Fig. 6e). Immunofluorescent staining of p-CREB and CREB in the knee joint sections. Scale bars, 50 μm. Dotted lines indicated the surface of articular cartilage. **k** The cAMP induced by HL-43 for 2 h in primary articular chondrocytes was measured, *n* = 3. Error bars are means ± s.d, *P < 0.05, **P < 0.01, ***P < 0.001, ****P < 0.0001 by one-way ANOVA with multiple comparison using Tukey method. ns not significant.

Next, we asked how *EP4* knockout or EP4 antagonist HL-43 promoted chondrogenic differentiation and cartilage anabolism. Our data so far had shown that knockout of *EP4*, or EP4 antagonist increased the expression of Col2 and Sox9 in human, rat and mouse cartilage (Figs. 2–6). Furthermore, Western blotting assay in primary mouse chondrocytes also confirmed that HL-43 dose-dependently induced Col2 expression (Fig. 7a). It is known that Sox9 regulates *Col2a1* expression through binding to a minimal 48 bp enhancer and promoting Col2a1 transcriptional activation^44^. We confirmed that HL-43 dose-dependently increased the activity of luciferase reporter (4 × 48-p89-Luc), which contains a reiterated Sox9 binding enhancer sequence (4 × 48) coupled to the mouse Col2a1 minimal promoter (p89) in the ATDC5 chondrocyte cells, suggesting HL-43 may regulate Col2a1 expression via Sox9 (Fig. 7b). The Sox9 promoter luciferase reporter (Sox9 promoter-Luc) activity also increased by HL-43 stimulation in a dose-dependent manner (Fig. 7c). After IL-1β incubation, Sox9 promoter activity was reduced, and HL-43 partially rescued the Sox9 transcriptional expression in a dose-dependent manner (Fig. 7d), suggesting that HL-43 could enhance the transcriptional expression of Sox9. Because the Sox9 promoter contained a transcriptional factor cyclic AMP response element binding protein (CREB) binding site^45^, we next examined whether HL-43 regulated Sox9 expression through cAMP/CREB signaling. Our data showed that HL-43 dose-dependently activated CREB luciferase reporter (CRE-Luc) under pro-inflammatory conditions (PEG2 or IL-1β) (Fig. 7e). Similar results were obtained by Western blotting that show that the phosphorylation of CREB under the pro-inflammation conditions was enhanced by HL-43 in a dose-dependent manner (Fig. 7f), and this CREB phosphorylation is dependent on EP4 (Fig. 7g).

We found that both HL-43 and Grapiprant can increase CRE-Luc activity in a dose dependent manner, with HL-43 having a better efficiency (Supplementary Fig. S9a). Furthermore, the inhibition of CREB upstream kinase PKA, by the selective inhibitor H89 completely abolished HL-43-induced CRE luciferase reporter and Sox9 promoter luciferase reporter activity (Fig. 7h, i). The upregulation of CREB phosphorylation was also detected in articular cartilage of mice treated with HL-43 following DMM surgery, compared with the vehicle treated group (Fig. 7j). Moreover, the cAMP assay showed that HL-43 dose-dependently induced cAMP production in the primary mouse chondrocytes (Fig. 7k). To further verify that HL-43 promotes chondrogenic differentiation and cartilage anabolism via Sox9 signaling, its target chondrogenesis and cartilage ECM related genes were analyzed by qRT-PCR. Our results confirmed that the Sox9 target genes including *Col2a1*, *Acan*, *Sox5*, *Col9a1*, *Col11a1*, *Col27a1*, *Dcn*, and *Prg4* were upregulated in ATDC5 cells treated with HL-43 (Supplementary Fig. S9b).

### EP4 antagonist HL-43 attenuates articular cartilage catabolism by downregulating of STAT3 signaling

**Fig. 8:**
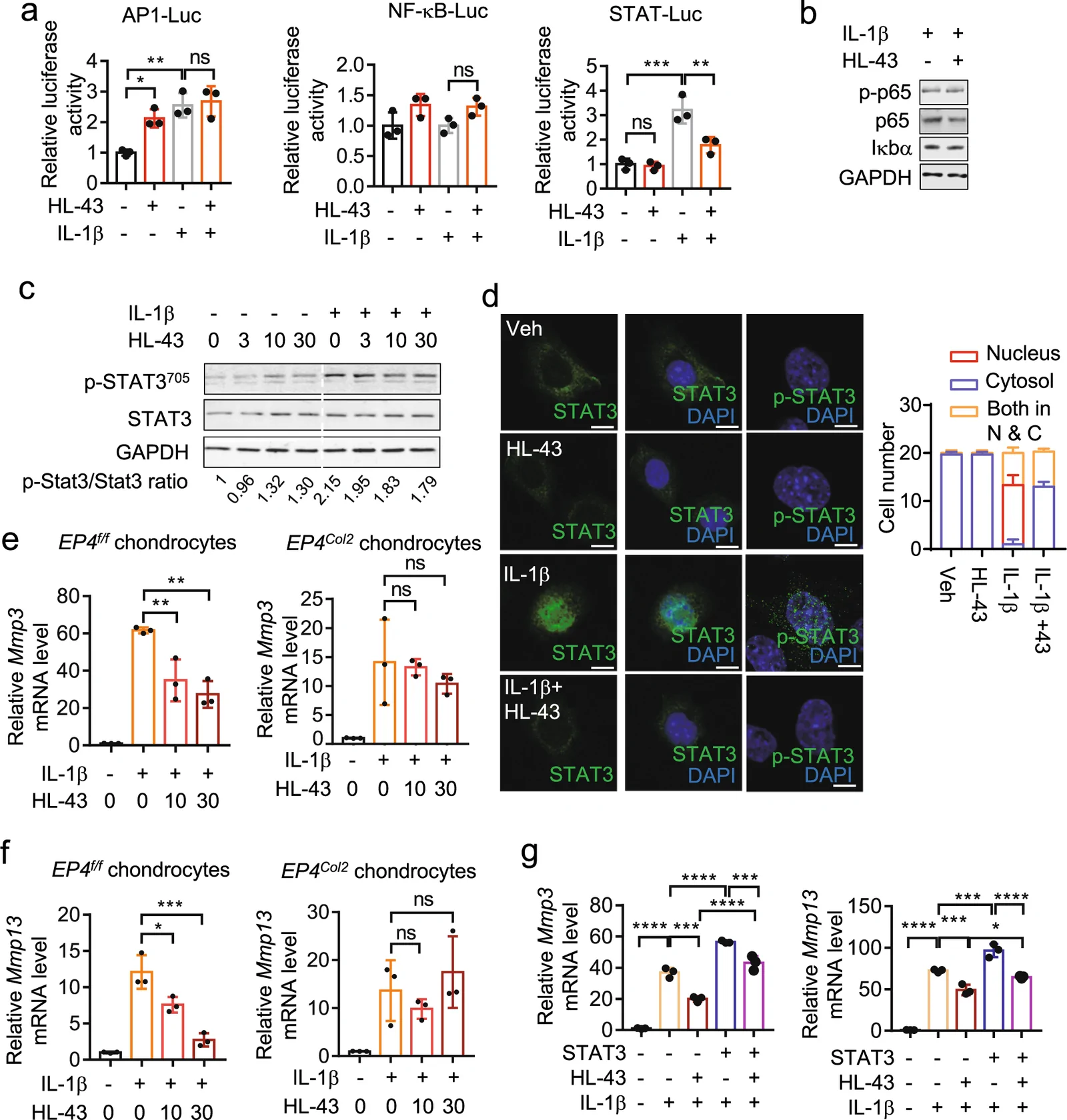
EP4 antagonist HL-43 suppressed cartilage catabolism through STAT3 signaling. **a** HL-43 inhibited STAT luciferase reporter activity, but not AP1 or NF-κB activity. After transfecting with the AP1-Luc, NF-κB-Luc, Stat-Luc reporter, the ATDC5 cells were treated with or without 2 ng/ml IL-1β and 30 μM HL-43 for 6 h. The cell lysates were subjected to Dual-Luciferase assays (*n* = 3). **b** Primary culture of mouse articular chondrocytes was treated with IL-1β and 30 μM of HL-43 for 1 h. p-P65, P65, IκBα protein levels were detected by Western blotting. **c** Primary culture of mouse articular chondrocytes was treated with or without 2 ng/ml IL-1β and the indicated concentrations (μM) of HL-43 for 1 h. p-STAT3^705^, STAT3, and GAPDH protein levels were detected by Western blotting. Protein bands in the blot were quantified by densitometry and analyzed using Image J. The numbers indicate grayscale ratios of p-STAT3^705^/STAT3. **d** HL-43 inhibited IL-1β-induced STAT3 and p-STAT3 nuclear translocation. Immunofluorescence staining of STAT3 and p-STAT3 subcellular localization of ATDC5 cells (left). Cells were treated with or without HL-43 (30 μM) and 5 ng/ml IL-1β for 24 h. Scale bars, 10 μm. Statistical analysis for STAT3 cellular distribution by confocal microscope (right). **e**, **f** HL-43 regulated expression of *Mmp3* and *Mmp13* in an EP4 dependent manner. qRT-PCR analysis of *Mmp3* (**e**)*, Mmp13* (**f**) expression in primary articular chondrocytes isolated from new born *EP4*^*f/f*^ or *EP4*^*Col2*^ mice that were treated with or without 2 ng/ml IL-1β and indicated concentrations (μM) of HL-43 for 24 h (*n* = 3). **g** qRT-PCR analyses of *Mmp3, Mmp13* expression in ATDC5 cells that transfected with or without STAT3 plasmids, then treated with or without 2 ng/ml IL-1β, 30 μM of HL-43 for 24 h. Unless otherwise stated, graphs show means ± s.d., *P < 0.05, **P < 0.01, ***P < 0.001, ****P < 0.0001 by one-way ANOVA with multiple comparison using Tukey method to compare between specific means.

To elucidate the effects and the intracellular signaling of HL-43 on catabolic factors such as MMP-3/13 expression in chondrocytes, we firstly examined the activity of AP-1, NF-κB, and STAT using luciferase reporters^46–48^. The data showed that the luciferase reporter activity of STAT, but not AP-1 or NF-κB was significantly inhibited by HL-43 under IL-1β stimulation (Fig. 8a). The PKA inhibitor H89 treatment did not alter this inhibitory effect, which suggests an absence of PKA signaling involvement. (Supplementary Fig. S9c). Western blotting also confirmed that HL-43 had little effects on the phosphorylation of p65, and degradation of IκBα (Fig. 8b), but reduced the phosphorylation of STAT3 (Fig. 8c). Furthermore, HL-43 also suppressed the IL-1β-induced nuclear translocation of endogenous STAT3 and p-STAT3 in chondrocytes (Fig. 8d). STAT3 can bind to the MMP3/13 promoter and positively regulate their expression^48^. Our data showed that HL-43 dose-dependently inhibited EP4 (Fig. 8e, f) and STAT3 dependent (Fig. 8g) increase in MMP3/13 expression.

## Discussion

Currently, there are no effective therapies for articular cartilage defects, making it an unmet clinical need. The role of EP4 receptor in cartilage homeostasis and regeneration remains unclear, especially due to a lack of in vivo evidence. Here, we show that inhibiting EP4 signaling promotes chondrogenesis, and stimulates anabolism while suppressing catabolism in the articular cartilage and thus contributes to tissue regeneration, and not fibrocartilage formation. Furthermore, we identified a novel EP4 antagonist HL-43, which promoted cartilage repair and regeneration in three different CD animal models and showed preferable cartilage regeneration efficacy compared to other FDA approved NSAIDs — Celecoxib and Grapiprant. Importantly, we also demonstrate that HL-43 regulated cartilage anabolism through Sox9 signaling, and inhibited the cartilage catabolism via STAT3 signaling. We, therefore, for the first time provide evidence to suggest that blocking the EP4 receptor is a promising therapeutic strategy for cartilage repair and regeneration.

It has been reported that among the four PGE2 receptors, only EP2 and EP4 have increased expression in the articular cartilage and the inflammed joint tissue^23,49^. PGE2 has a stronger binding affinity to EP4 compared to EP2^37,50^. However, the role of EP4 in chondrocyte differentiation and cartilage catabolism is controversial. It has been reported that EP4 agonists can promote chondrocyte differentiation^51^, induce proteoglycan accumulation^24^, inhibit chondrocyte catabolism^26^, and thus ameliorate OA progression^36^; while EP4 antagonist can block chondrocyte anabolism^24^. However, other reports have shown that EP4 antagonists inhibit the gene expression of human and mouse catabolic chondrocyte markers in vitro^23,25^ and alleviate OA progression^31^. These contrary reports could be due to off-target effects of the small molecules used in these studies, and the lack of validation using *EP4* knockout models in vitro and in vivo. In our study, we found that both *EP4* knockout, and our novel EP4 antagonist HL-43 treatment promoted cartilage regeneration by enhancing chondrogenesis and cartilage anabolism, and suppressing chondrocyte catabolism. Furthermore, the lack of effect of HL-43 on *EP4* knockout cells validates our finding and gives us confidence that that EP4 is a negative regulator of cartilage homeostasis.

NSAIDs including COX-2 inhibitor are the first-line drug for reducing pain and inflammation for multiple indications including arthritis, and acute pains^52^. Although nine of the fourteen independent pre-clinical animal studies demonstrated chondroprotective effects for selective COX-2 inhibitors in OA, five clinical studies failed to demonstrate any chondroprotective actions in OA patients^52^. This highlights the limitations of using COX-2 inhibitors for articular cartilage repair and regeneration. In our DMM surgery-induced and aging-induced CD murine models of OA, we observed that Celecoxib, a COX-2 inhibitor, was chondroprotective and inhibited cartilage catabolism, but had little effect on promoting chondrocyte differentiation or ECM generation in human articular cartilage explants. In contrast, HL-43 effectively induced cartilage regeneration in our three different CD animal models, as well as in human articular cartilage explants, by inhibiting cartilage catabolism and promoting anabolism. This indicates that targeting the EP4 receptor has better efficacy on cartilage repair and regeneration compared to targeting the upstream molecule COX-2. Furthermore, COX-2 inhibitors are associated with a risk of fetotoxicity, cardiovascular, renal, gastrointestinal, and hepatic toxicity, which lead to a FDA warning^53,54^. In our study, we confirmed that our EP4 antagonist HL-43 had lower toxicity in chondrocytes (Fig. 4b), embryos (Supplementary Fig. S5d) and gastrointestinal tract (data not shown), compared to Celecoxib. Thus, the results suggest that EP4 is a promising therapeutic target for overcoming the side effects of COX-2 inhibitor and promoting cartilage regeneration.

Furthermore, compared to the other EP4 antagonist Grapiprant (FDA approved for dog OA pain), HL-43 had a better efficacy on cartilage repair and regeneration in MF surgery-induced CD rat model and DMM surgery-induced CD mouse model. It also had a preferable effect on promoting chondrocyte differentiation and ECM generation, and inhibiting matrix degradation in human articular cartilage explants. Therefore, HL-43 may be a more suitable candidate molecule to inhibit EP4 and promote cartilage repair. It is also becoming evident that EP4 signaling associates with tumor progression^20^, and Grapiprant is currently been used in clinical trials for colorectal cancer and lung cancer (www.ClinicalTrials.gov). HL-43, therefore, has the potential to be applied to other diseases where the role of EP4 is implicated, including cancer.

Clinically in OA patients, a widely used cartilage repair strategy is to regenerate cartilage through the MF surgery, in which surgeons drill into the osteochondral surface till the marrow cavity^7^. However, most of the regenerated cartilage by this method is fibrocartilage with inferior mechanical properties, and is easily worn compared to the normal stable mature articular cartilage. In our study, we show that in MF surgery-induced CD rat and mouse models, genetic or pharmaceutical inhibition of EP4 induced formation of stable mature articular cartilage, instead of fibrocartilage. Moreover, inhibition of EP4 also promoted cartilage repair in a DMM surgery-induced CD mouse model, accompanied with a reduction in inflammation and pain. Therefore, HL-43 administration either orally or in an intra-articular hydrogel injection after microfracture surgery could be a promising therapeutic strategy for OA cartilage regeneration and symptomatic improvement. It could also be employed in combination with other intra-articular injected drugs such as sodium hyaluronate and glucocorticoids, or the lubricating, anti-inflammation drug chitosan.

PGE2 has been reported to have an important role in tissue maintenance and regeneration. Upregulating the PGE2 levels by inhibition of prostaglandin-degrading enzyme 15-PGDH or activation of COX-2 promotes hematopoiesis, liver and colon regeneration, rejuvenates aged muscle mass, and strength in mice^55–59^. Similarly, activation of COX-2 accelerates the bone fracture repair process by augmenting osteoblast differentiation, and suppressing chondrocyte differentiation^60^. It has been reported that sensory nerves perceive the concentration of PGE2 in response to changes in bone density, mechanical stress, and the metabolic activity that controls bone homeostasis^30^. Moreover, deletion of EP4 in these sensory nerves significantly reduced bone volume in adult mice. Furthermore, SW033291, a small molecule that increases PGE2 levels locally, significantly boosted bone formation and liver regeneration, whereas the effect was obstructed in the sensory nerve-specific *EP4* knockout mice^30,61^. However, as articular cartilage bears no nerve fibers and blood vessels, the PGE2/EP4 regulation in articular cartilage chondrocytes apparently differs from bone and other tissues. The enhanced chondrocyte differentiation and inhibition of chondrocyte hypertrophy observed here suggests that PGE2 signaling may be tissue specific, and is a negative regulator of cartilage regeneration. The four PGE2 receptors further adds to the complexity of PGE2 signaling and may play a distinct roles in tissue regeneration.

Besides PGE2, multiple ligands have been reported to be able to activate EP4, including PGF2α and PGE1^50,62^. PGF2α was shown to be involved in skeletal muscle regeneration^63^, while PGE1 regulates the axonal regeneration^64^, peripheral nerve regeneration^65^, and prevents liver failure by stimulating hepatocyte proliferation^66^. The roles of these ligands in EP4 mediated effects on the cartilage remain to be elucidated.

In conclusion, this study unveils a role for EP4 mediated cartilage repair and regeneration. We show that the novel EP4 antagonist HL-43 modulates cartilage hemostasis by enhancing cartilage anabolism through Sox9 signaling and inhibiting cartilage catabolism via STAT3 signaling, resulting in induction of normal stable mature articular cartilage instead of fibrocartilage. Our findings demonstrate that EP4 can act as a promising therapeutic target for cartilage regeneration, and the novel antagonist HL-43 has the clinical potential to be used for cartilage repair and regeneration.

## Materials and methods

### Collection and preparation of human articular cartilage explants

Human articular cartilage explants of OA patients undergoing total knee replacement surgery were collected (3 mm × 3 mm), with the approval of the Human Ethics Committee of Shanghai Sixth People’s Hospital (2021-048). The full thickness cartilage tissue samples were harvested from tibia plateau avoiding subchondral bone. We control the quality of the explants according to the principle mentioned below. (1) The human articular cartilage explants were harvested from the relatively intact region of the tibia plateau. (2) The explants from different patients were cut from the same position of tibia plateau avoiding subchondral bone. (3) The explants were cut in the size of 3 mm × 3 mm for the DMMB assay and the S.O. staining. (4) To avoid inter-patient and intra-patient sample heterogeneous, explants used within the same drug experiment (vehicle and treatment) must be collected in locations in close proximity and from the same patient. Each explant in the groups that treated with the same drug was from the same patient. The cartilage explants for Safranin-O/Fast-green (S.O.) staining and immunofluorescence were fixed in 4% paraformaldehyde (PFA) (wt/vol) overnight at 4 °C, embedded in paraffin, sliced (7 μm) and mounted on positively charged slides. The cartilage explants (3 mm × 3 mm) for DMMB assay and S.O. staining were harvested and placed in 24-well plates and cultured in 1 ml of serum-free F12 culture media for 24 h. *n* ≥ 4 patients were assessed. The media was then replaced with F12 culture media containing serum, added 1 ng/ml IL-1β, Celecoxib, Grapiprant, HL-43, and explants were cultured for a further 7 days. The culture media was collected for the release of glycosaminoglycans. To quantify GAGs, media were mixed with DMMB in formate buffer and absorbance at 535 nm was determined using a Spectramax plate reader (Molecular Devices, Sunnyvale, CA). The ratio between the secreted GAG was normalized to the weight of the cartilage explant to calculate the release of GAG. Safranin O-fast green staining was used to assess the percentage area of proteoglycans in the explant sections. We calculated the proteoglycan staining area using Image J with a region of interest of a 1 × 1 mm square, drawn at the edge of the cartilage surface under 10× objective. The data was presented as proteoglycan stained area/total cartilage explant area in the selected region.

### Mice and experimental surgery

*EP4*^*f/f*^ mice^67^ were crossed with the *Col2-cre* strain to generate *Col2-cre; EP4*^*f/f*^ (*EP4*^*Col2*^) mice, or were crossed with *Aggrecan-creERT2*^68^ mice to generate *EP4*^*f/f*^; *Aggrecan-CreERT2* (*EP4*^*AcanERT2*^) mice. Mice were maintained at the animal center of East China Normal University. All mouse experiments were approved by Ethics Committee at East China Normal University. Mice of the same genetic background were generated and raised under identical conditions. Sex-matched littermate mice were compared. For the DMM surgery-induced CD mouse model, WT mice (strain C57/BL/6) or *EP4*^*Acan*^ mice were used, *n* = 5 for each group. DMM surgery was carried out as previous described using 10-week-old male mice^69,70^; while Sham-operated mice were used as controls. Knee joints were collected for histological analysis 6 or 8 weeks after surgery. Osteoarthritis Research Society International (OARSI) histopathology grading system was used to evaluate the sections^71^, six sections of the articular cartilage of each mouse were evaluated. Synovitis was assessed by the synovitis score^72^. Therapy with oral EP4 inhibitors was initiated one day after surgery and continued for 8 weeks, *n* = 8 for each group. EP4 inhibitors and Celecoxib were dissolved in carboxymethylcellulose sodium which is prepared at the concentration of 0.5%, and vehicle contained carboxymethylcellulose sodium alone. The vehicle, Celecoxib, Grapiprant, and HL-43 were delivered to mice at indicated doses daily by gavage, Celecoxib purchased from damas-beta (29322B), Grapiprant was purchased from Yuduobio, Shanghai, all other EP4 antagonists were synthesized by Dr. Hankun Zhang’s lab.

### Microfracture (MF) surgery-induced cartilage defect (CD) mouse model

Six-week-old male littermates of *EP4*^*f/f*^ and *Ep4*^*Col2*^ mice were used to detect the regeneration ability of articular cartilage after MF surgery. We use a 26G needle covered with a sheath as a manual drill to create cartilage defect, the sheath is 500 μm shorter than the 26G needle. Under general anesthesia, a medial parapatellar incision was made to expose the femoral condyles. Full-thickness articular cartilage injuries were generated in the right femur patella groove; the left femur underwent sham operation which exposes the patella but without damaging the cartilage. The patella was repositioned and joint capsule and skin were sutured separately. After 2 weeks (*n* = 3 for each group), 4 weeks (*n* = 7 for each group) and 8 weeks (*n* = 5 for each group), the repaired articular cartilage was histologically analyzed and correlation scored.

### Aging-induced cartilage defect (CD) mouse model

WT male mice (strain C57/BL/6) were raised under identical conditions until 19 months. The vehicle, Celecoxib, and HL-43 were administered to mice at 30 mg/kg daily by gavage from 19 to 22 month, *n* = 9 for vehicle and HL-43-treated groups, *n* = 6 for Celecoxib treated group.

### Preliminary pharmacokinetic studies of HL-43 and HL-66 in mouse model

To evaluate the preclinical pharmacokinetic (PK) profiles of compound HL-43 and HL-66, 36 male CD1 mice were randomly divided into four groups for PK studies. The test compounds were administrated intravenously (I.V.) or orally by gavage (P.O.) at the dosage of 1 or 5 mg/kg to the mice, respectively. Blood samples were collected at 0.083, 0.25, 0.5, 1, 2, 4, 8, 12, and 24 h after administration (*n* = 3). Then the whole blood was centrifuged at 5500 × *g* for 15 min at 4 °C. After suitable sample preparation, the concentration of HL-43 and HL-66 in plasma sample was determined by Agilent HPLC-MS/MS system (Agilent Technologies, USA). Pharmacokinetic parameters were calculated by WinNonlin software version 7.0 based on non-compartmental analysis (Pharsight Corporation, Mountain View, USA). Mean plasma concentration-time curves were plotted by GraphPad Prism 7.0 (GraphPad Software Inc., CA, USA).

### Preparation of starch hydrogel and miscibility of HL-43 in 14 Gel

Ca(NO_3_)_2_·4H_2_O was obtained from Sinopharm Chemical Reagent. Waxy starch (amylopectin more than 90%) was purchased from Qinhuangdao Lihua Starch. For the preparation of starch hydrogels, a fixed water-to-starch mass ratio of 5 was used, and the crosslinker Ca(NO_3_)_2_·4H_2_O was dissolved in the starch solution. The mixture was stirred at 60 °C for 1 h to obtain a viscous gel. The starch hydrogel prepared with Ca(NO_3_)_2_·4H_2_O and Waxy starch was named as 14 Gel. The hydrogel is sterilized by UV for 30 min and then used. HL-43 and Grapiprant were each dissolved in the DMSO solvent (100 mM). Compound/14 Gel was prepared by mixing the solutions at a weight ratio of 1/10 (w/w).

### Microfracture (MF) surgery-induced cartilage defect (CD) rat model

Thirty-six male Sprague-Dawley (SD) rats (280–300 g) were randomized into three groups (each group *n* = 4 rats for each time point), Sham-operated mice were used as controls (*n* = 4). After anesthetizing with 3.5% chloral hydrate (10 ml/kg), the rats were fixed on the operating table in supine position and the skin was prepared and disinfected. A 2 cm longitudinal incision along the medial margin of the patella of the right knee was made with a scalpel and then the capsule was open to expose the articular cartilage surface. A manual drill with a diameter of 2 mm was used to drill holes in the femoral intercondylar fossa of the rat (2 mm × 2 mm). After washing with saline, the gauze was pressed and 100 μl 14 Gel containing appropriate drugs or vehicle were injected. After the injection, the compression flexes and compresses were applied several times to spread the gel throughout the knee joint. The ligaments and skin were sutured. After 2, 4, and 6 weeks, the repaired articular cartilage was histologically analyzed and correlation was scored.

### Zebrafish embryo toxicity assay

A breeding stock of zebrafish at the age of 6–24 months is used for egg production. Fertilized eggs were collected and viable eggs were transferred to the 12-well plate at 26 °C. Fertilized eggs between the 4-cell and 128-cell stages be used for the toxicity test, with ten fertilized eggs per concentration and three replicates of each concentration, a total of 30 eggs for each concentration. Grapiprant and HL-43 were tested in three concentrations (3 mM, 1 mM, 300 μM), and Celecoxib were test in four concentrations (1 mM, 300 μM, 100 μM, and 30 μM) which were lower than Grapiprant and HL-43 due to its high toxicity. DMSO was added as vehicle control. Hatching, morphological abnormalities and survival were assessed 24 and 72 h of post-fertilization (dpf) in zebrafish exposed to the chemicals.

### Mechanical threshold sensory test

Von Frey nylon filaments were used to test the hind-paw reflex sensitivity to punctate static mechanical stimuli. Mice were habituated to the wire mesh bottom cages for 30 min before the test. von Frey filaments (Aesthesio von Frey Kit, Ugo Basile, Varese, Italy) were applied from underneath the mouse in ascending order to the mid-plantar surface of the hind paws. If withdrawal response was not induced, the next level of von Frey hair was applied until at least six withdrawals occurred in ten stimuli. This minimal force required to elicit 50% positive response was recorded as the paw withdrawal threshold^73,74^.

### Measurements of thermal hyperalgesia

The paw withdrawal latency (PWL) and threshold (PWT) of thermal hyperalgesia were measured using the Ugo-Basile 37370 plantar test (Comerio, Italy). The mice were placed on a glass plate of constant temperature for 30 min before each test. Thermal thresholds of the ipsilateral hind paws were assessed three times with a 10 min interval between trials. The time(s) from irradiation to paw withdrawal was recorded as the thermal withdrawal latency. The intensity of the infrared generator was adjusted to produce withdrawal latencies of approximately 10 s (25 infrared intensity).

### Hind-limb weight bearing analysis

Osteoarthritic pain can be caused uneven weight-bearing. Hindlimb weight bearing analysis was used to measure DMM induced OA pain in mice^75^. The weight borne by the DMM-operated hindlimb was compared to the non-operated contralateral hindlimb. An incapacitance tester (YLS-11A, Yi Yan Technology Development Co., Ltd., Jinan, China) was used for calculating hindlimb weight distribution. Mice were trained to stand upright with hindlimbs on a separate force plate. Weight-bearing load is calculated by amount of weight (g) of un-operated contralateral control − amount of weight (g) of the operated limb.

### Culture of mice cartilage explants

WT mice at the age of 10 weeks were used for isolating femoral head cartilage explants. The femoral heads were dissected and submerged into F12 medium. The femoral head cartilage explants for dimethylmethylene blue (DMMB) assay were placed in 24-well plates and cultured in 1 ml of serum-free F12 culture media for 24 h. Culture media were then replaced with serum containing medium with 1 ng/ml IL-1β, Celecoxib, Grapiprant, and HL-43 for 48 h. The culture media was collected for the evaluation of the glycosaminoglycans release.

### Atomic force microscopy

Eight weeks of posts MF surgery, the mice were euthanized and distal femurs were dissected. Utilizing a microtome blade to bisect the defect region. Half of the tissue was immediately fixed in 4% PFA and then embedded in paraffin. The other half of the fresh tissue was dissected further and cleaned. The tissue was then fixed to a plate utilizing tissue glue, and the plate was filled with protease-free PBS. The samples were imaged on a Bruker FastScan Bio-AFM. We used a pre-calibrated set of Nanotools 0.10 N/m 12 μm MLCT-O10 biosphere spherical probes. All variables were kept constant throughout experimental groups. The NanoScope Analysis 1.8 Software was used for analysis.

### Cell viability

The cell viability was evaluated by CCK-8 assay (Dojindo Molecular Technologies). Primary chondrocytes were plated in the 96-well plates and treated with the EP4 antagonists or DMSO for 72 h. A total of 10 μl CCK-8 was added to each well, the cells were incubated in the presence of CCK-8 for 2–3 h. The optical density (OD) of each well at 450 nm was recorded on a Microplate Reader (SPECTRA MAX190). The absorbance values were normalized by subtracting blank values of untreated cells. The cell viability (% of control) is calculated as the percentage of (OD_test_ − OD_blank_)/(OD_control_ − OD_blank_). The 50% inhibitory concentration (IC_50_) was calculated with GraphPad Prism software using the sigmoidal dose-response function.

### Cell growth assayed by SRB assay

Cell growth was determined by the sulforhodamine B (SRB) assay^76^. Primary articular chondrocytes were isolated from *EP4*^*Col2*^ and *EP4*^*f/f*^ knee joints of newborn pups. Cells were seeded at 2000/well in 96-well culture plates, following 0, 2, 4, and 6 days incubation period in DMEM/F12 medium. Cells were fixed and stained with SRB (0.4% SRB in 1% CH_3_COOH) for 10 min at room temperature. Washing cells with 1% CH3COOH until clear. Adding the Tris base solubilized SRB dye. Absorbances were measured using a spectrophotometer at 510 nm with a 630 nm background and all data at day 2, 4, and 6 were normalized to the data at day 0.

### Cell line and chondrocyte primary culture and differentiation

ATDC5 cell line was purchased from Riken BioResource Center (Tsukuba, Japan) and grown in a 1:1 mixture of DMEM/F12 medium (Corning, 10-092-CVR) supplemented with 5% fetal bovine serum (Gibco, 10099, USA) and 1% penicillin-streptomycin solution (Life Technologies). Cells were maintained at 37 °C under 5% CO_2_. Primary chondrocytes were obtained from knee joint cartilage of newborn mice. Knee joints were dissected from the tibia plateau and femoral condyle articular cartilage of mouse pups and digested in 0.1% collagenase type II (Sigma) overnight at 37 °C and chondrocytes were harvested by centrifugation. Primary chondrocytes were cultured at a density of 5 × 10^3^ cells per ml at 37 °C with 5% CO_2_. DMEM/F12 medium with 10% fetal bovine serum (FBS) and 1% penicillin–streptomycin was used to culture the primary chondrocytes. Chondrogenic differentiation were induced with chondrogenic medium, which contains insulin-transferrin-selenium (ITS) (Sigma, I3146), 10 ng/ml TGF-β3 (PEPROTECH, 100-36E, USA), 100 nM Dexamethasone (Sigma, D4902), 50 μg/ml VC (Sigma), 1 mM sodium pyruvate (Sigma) and 40 μg/ml proline (Sigma), and/or treatment with rmIL-1β (R&D, 401-ML/CF), TNFα (PEPROTECH, 300-01A-50) in DMEM/F12 for indicated days. Western blotting was performed according to standard protocols. Antibodies used were: rabbit anti-Mmp3 (1:1000, HuaBio), rabbit anti-Mmp13 (1:500, abcam, ab39012), rabbit anti-p-CREB (1:2000, Cell Signaling Technology, 87G3), rabbit anti-CREB (1:2000, Cell Signaling Technology, 48H2), mouse anti-GAPDH (1:5000; Sigma-Aldrich, G8795), rabbit anti-Col2 (Abcam, Ab34712), rabbit anti-STAT3 (1:2000; Abcam, ab68153), rabbit anti-p-STAT3 (Y705) (1:2000; Abcam, ab76315), rabbit anti -p65 (1:1000; CST, 3034), rabbit anti-p-p65 (1:1000; CST, 3033), and rabbit anti-IκBα (1:1000; Abways, CY5026).

### cAMP assay

The cAMP concentration of primary chondrocytes was detected by cAMP dynamic kit (Cisbio, France) following the manufacturer’s instructions. cAMP standards were diluted according to the kit instructions. Cells were treated with HL-43 for 2 h and the relative fold change of cAMP was determined by comparing with the cAMP standard curve.

### Quantitative RT-PCR

Quantitative RT-PCR was performed to measure the relative mRNA levels. RNA samples were prepared from the chondrocytes using Trizol (Takara). cDNA was generated using the Prime Script RT reverse transcriptase kit (Yeasen, Drr036a). Primers used for amplification were shown in Supplementary Table S1, GAPDH was used as the house keeping gene.

### Luciferase assay

Six firefly luciferase reporters were used in the Luciferase assay, including CRE-Luc (PathDetect CRE cis-Reporting System; Stratagene, La Jolla CA) driven by a basic promoter (TATA box) plus four repeats of CRE; Sox9 promoter-Luc contains human SOX9 proximal promoter region (−1034 bp to +67 bp)^45^; 4 × 48-p89-Luc, mouse Col2a1 minimal promoter^44^. AP-1-Luc, Nf-κb-Luc, STAT-Luc were obtained from Stratagene. Chondrocytes were cultured in 24-well plates and 300 ng firefly luciferase reporter constructs were transiently transfected using transfection reagent TransExellent-DNA (Cenji Biotech Inc, China) following manufacturer’s instruction, and 15 ng of the Copia-Renilla luciferase reporter was co-transfected as a normalization control. Cells were treated with the indicated reagents for 48 h after transfection. The Dual-Luciferase measurements were performed in triplicate using the Luciferase assay kit (Promega) and a LUMIstar Omega luminometer (BMG Labtech, Mornington, Australia).

### Immunohistochemistry

Knee joints of mice were fixed for 24 h in 4% paraformaldehyde (wt/vol), dehydrated into 100% ethanol, and decalcified for 1 week in 0.5 M EDTA pH 8.0. Paraffin-embedded tissue was sectioned (7 μm) and stained with Safranin O and Hematoxylin & Eosin (H&E) according to standard procedures. The OARSI histologic scoring of OA in the mouse was used to measure the cartilage and synovial damage of OA^71,77^. Immunohistochemical staining for Col2a1, ColX, Sox9, p-CREB, and CREB were routinely performed and follow previous description^78^. The primary antibodies used were, rabbit anti-Col2 (Abcam, Ab34712), rabbit anti-Acan (1:100, Proteintech, 13880-1-AP), rabbit anti-ColX (1: 1000, Abcam; ab58632), rabbit anti-Mmp13 (1:500, abcam, ab39012), rabbit anti-Sox9 (1;500, EMD Millipore; ab5535), rabbit anti-p-CREB (1:2000, Cell Signaling Technology, 87G3), rabbit anti-CREB (1:2000, Santa Cruz Biotechnology, sc-25785), rabbit anti-STAT3 (1:200; Abcam, ab68153), rabbit anti-p-STAT3 (Y705) (1:200; Abcam, ab76315), mouse anti-Col1 (1:200; Santa Cruz, sc-59772), mouse anti-EP4 antibody (1:500, Santa Cruz, sc-55596). Images were taken on the Olympus BX53 microscope, Olympus SZX16 microscope or Leica SP8 confocal laser scanning microscope. Histomorphometry and quantification were performed using ImageJ software. Quantification of Mmp13, ColX, Sox9 positive regions were quantified as the positive area/total cartilage area per field of view under ×40 objective magnification.

### Alcian blue staining

Micromass cultures were rinsed with PBS, fixed in PFA for 10 min, and then incubated with Alcian blue staining solution (1.0% Alcian blue in 0.1 N HCl) overnight at 4 °C. Excess stain was washed off with ddH_2_O. Alcian blue-stained cultures were extracted using 6 M guanidine hydrochloride and absorbance was measured at 620 nm.

### BMSC isolation and adenovirus cell culture treatment

The bone marrow cavity of 6–8-week-old *EP4*^*f/f*^ mice were flushed to isolate BMSCs. The flushed cells were cultured in modified Alpha-MEM, 10% FBS, 1% P/S and 24 h later, the petri dish was washed with PBS to remove mononuclear macrophages. The isolated BMSCs were seeded in 24-well plate at 6 × 10^4^ cells per well. After the cells attached to the dish, we used HBAD-CRE adenovirus (HANBIO, HH20210508LCH-AP01) and HBAD-EGFP (HANBIO, HH20210510LCH-AP01) as controls to transduce the cells. The MOI used was 300. After 2 days of viral transduction, chondrogenic differentiation was induced in chondrogenic media, and the knockout efficiency and the expression of chondrogenic marker genes was assessed after 16 days using RT-qPCR. Alcian blue staining was also performed to detect the chondrogenic differentiation.

### Statistical analysis

GraphPad Prism seven (GraphPad, San Diego) was used for statistical analysis. One-way ANOVA with Tukey’s multiple comparisons was used to compare the groups. Two-tailed Student’s *t*-test was used to detect statistically significant treatment effects when only two groups were compared. Two-way ANOVA with multiple comparisons test using the Sidak’s test was used to comparing groups in cell proliferation assay. *P* < 0.05 were considered as statistically significant (*P < 0.05, **P < 0.01, ***P < 0.001). Quantification was performed from at least three independent experimental groups and presented as mean ± s.d.

## Supplementary information

Supplemental information
