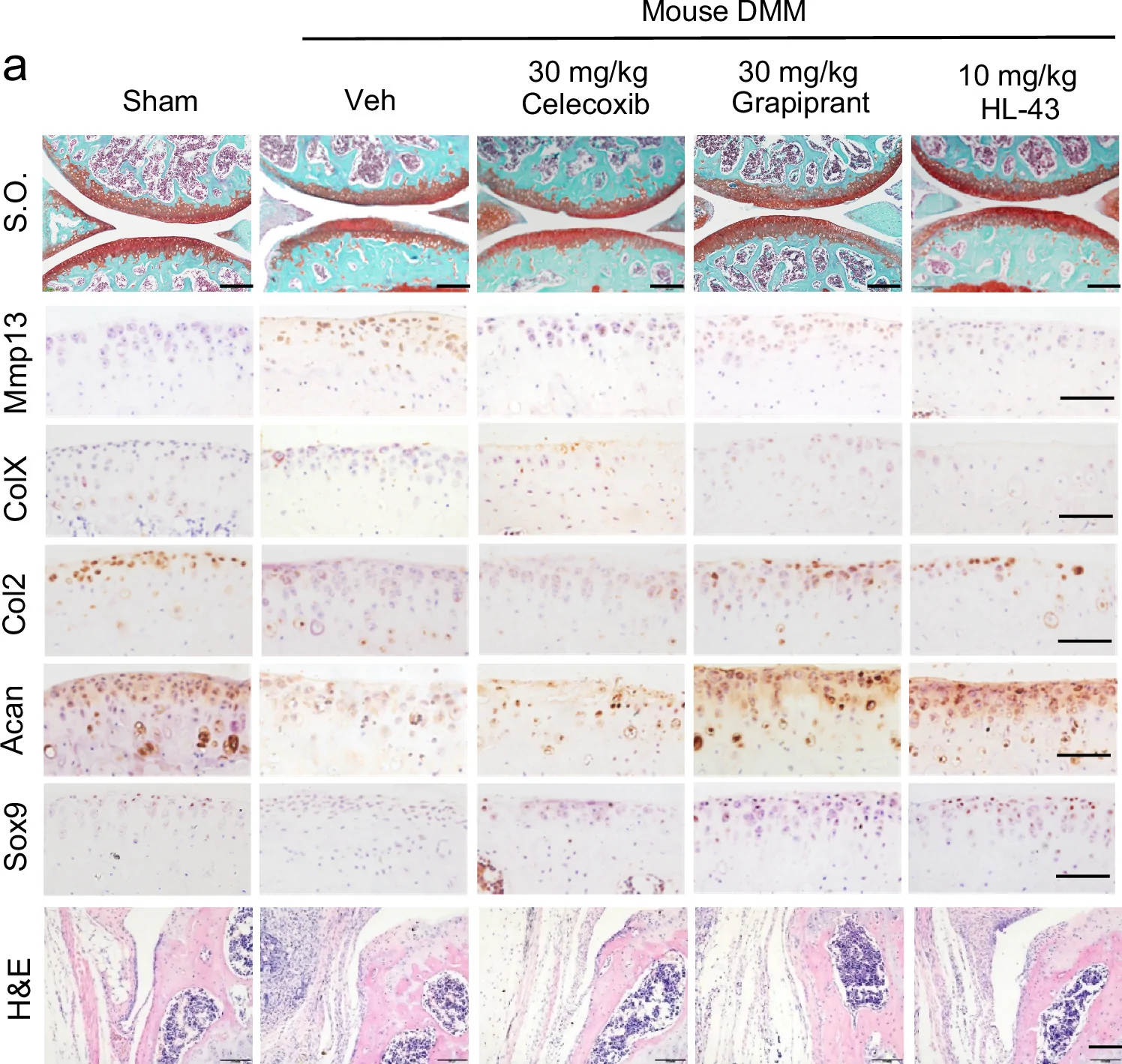# Author Correction: A novel prostaglandin E receptor 4 (EP4) small molecule antagonist induces articular cartilage regeneration

**DOI:** 10.1038/s41421-026-00933-1

**Published:** 2026-10-13

**Authors:** Yunyun Jin, Qianqian Liu, Peng Chen, Siyuan Zhao, Wenhao Jiang, Fanhua Wang, Peng Li, Yuanjin Zhang, Weiqiang Lu, Tao P. Zhong, Xinran Ma, Xin Wang, Alison Gartland, Ning Wang, Karan Mehul Shah, Hankun Zhang, Xu Cao, Lei Yang, Mingyao Liu, Jian Luo

**Affiliations:** 1 Shanghai Key Laboratory of Regulatory Biology, Institute of Biomedical Sciences and School of Life Sciences, East China Normal University, Shanghai, China; 2 Yangzhi Rehabilitation Hospital (Sunshine Rehabilitation Centre), Tongji University School of Medicine, Shanghai, China; 3 Orthopedic Institute, Soochow University, Suzhou, Jiangsu China; 4 Department of Oncology and Metabolism, The University of Sheffield, Sheffield, UK; 5 Departments of Orthopaedic Surgery and Biomedical Engineering and Institute of Cell Engineering, The Johns Hopkins University School of Medicine, Baltimore, MD USA; 6 Center for Health Science and Engineering, School of Materials Science and Engineering, Hebei University of Technology, Tianjin, China

Correction to: *Cell Discovery* https://doi.org/10.1038/s41421-022-00382-6 published online 08 March 2022

During a recent review of our published paper, we identified two inadvertent errors in Fig. 5a and Supplementary Fig. S8a:

**In Fig. 5a:**
  The “vehicle (Veh)” image for HL‑43 is incorrectly identical to the “Veh” image for Celecoxib.
  The image intended for the “10 μM HL‑43” treatment group was mistakenly taken from the “30 μM HL‑43” treatment group.
**In Supplementary Fig. S8a:**
  The image showing Sox9 staining in the Sham group is mistakenly identical to the image showing Mmp13 staining in the 10 mg/kg HL‑43 group.

The images for Fig. 5a (vehicle and 10 μM in HL-43 treated group) and Supplementary Fig. S8a (Sox9 in sham group) have been corrected. This correction does not affect the results or the conclusion of this work. We sincerely apologize for any inconvenience that may have been caused by our error.

**Incorrect Figure 5**

**Figure:**
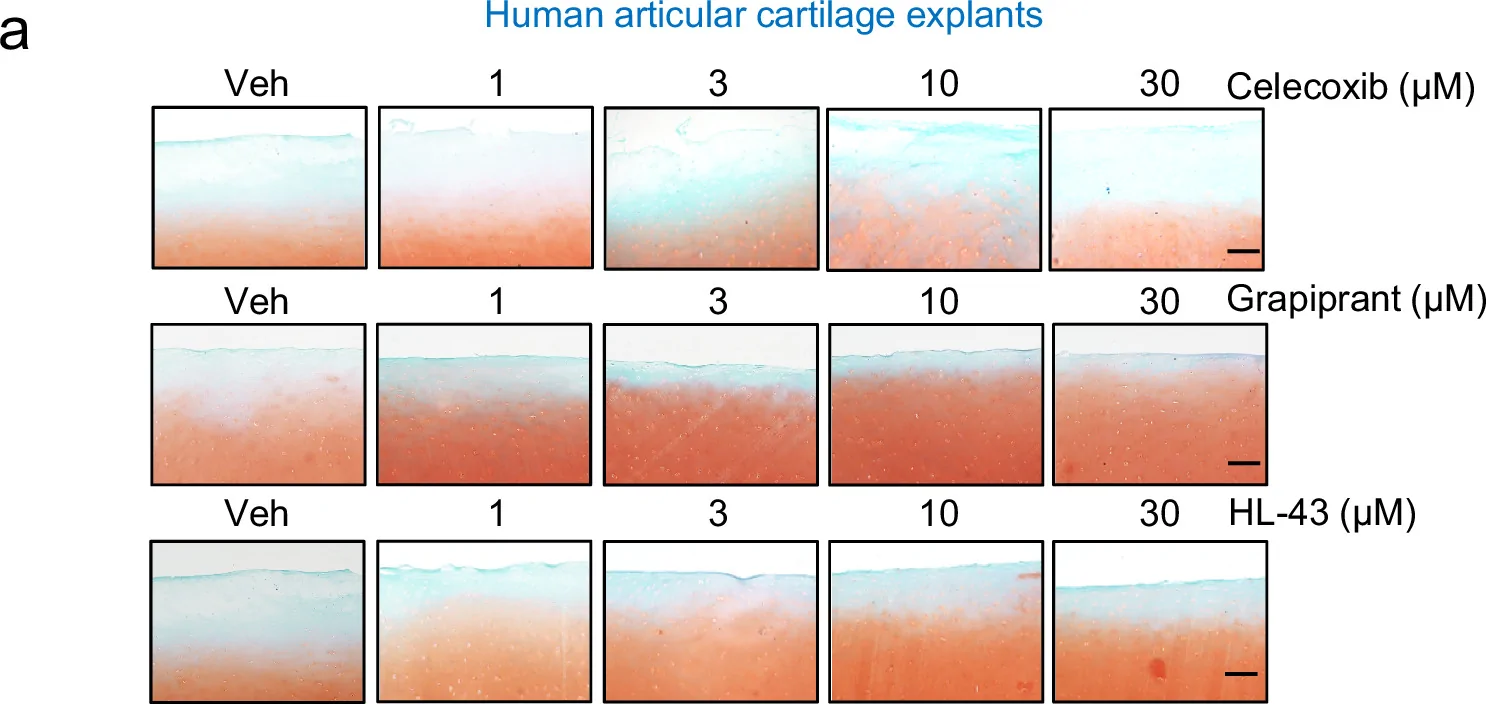

**Correct Figure 5**

**Figure:**
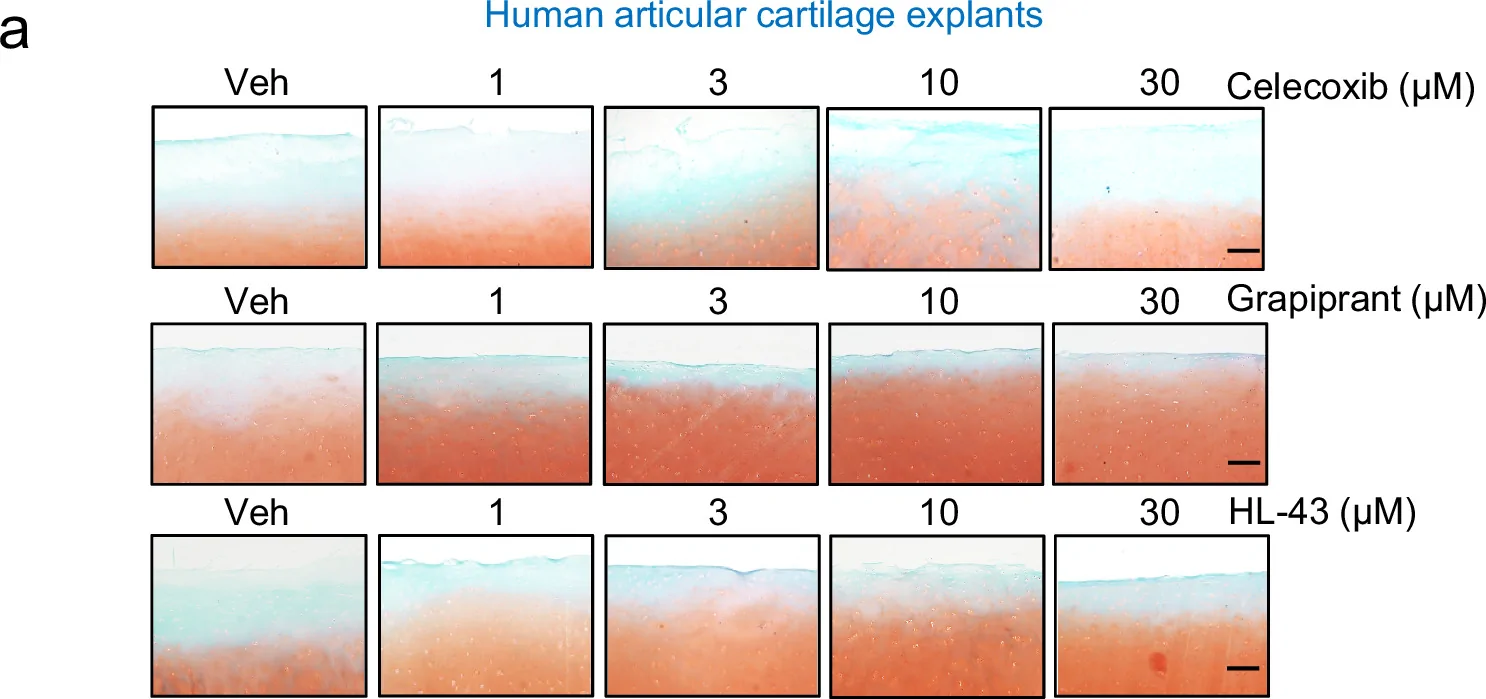

**Incorrect Supplementary Figure 8**

**Figure:**
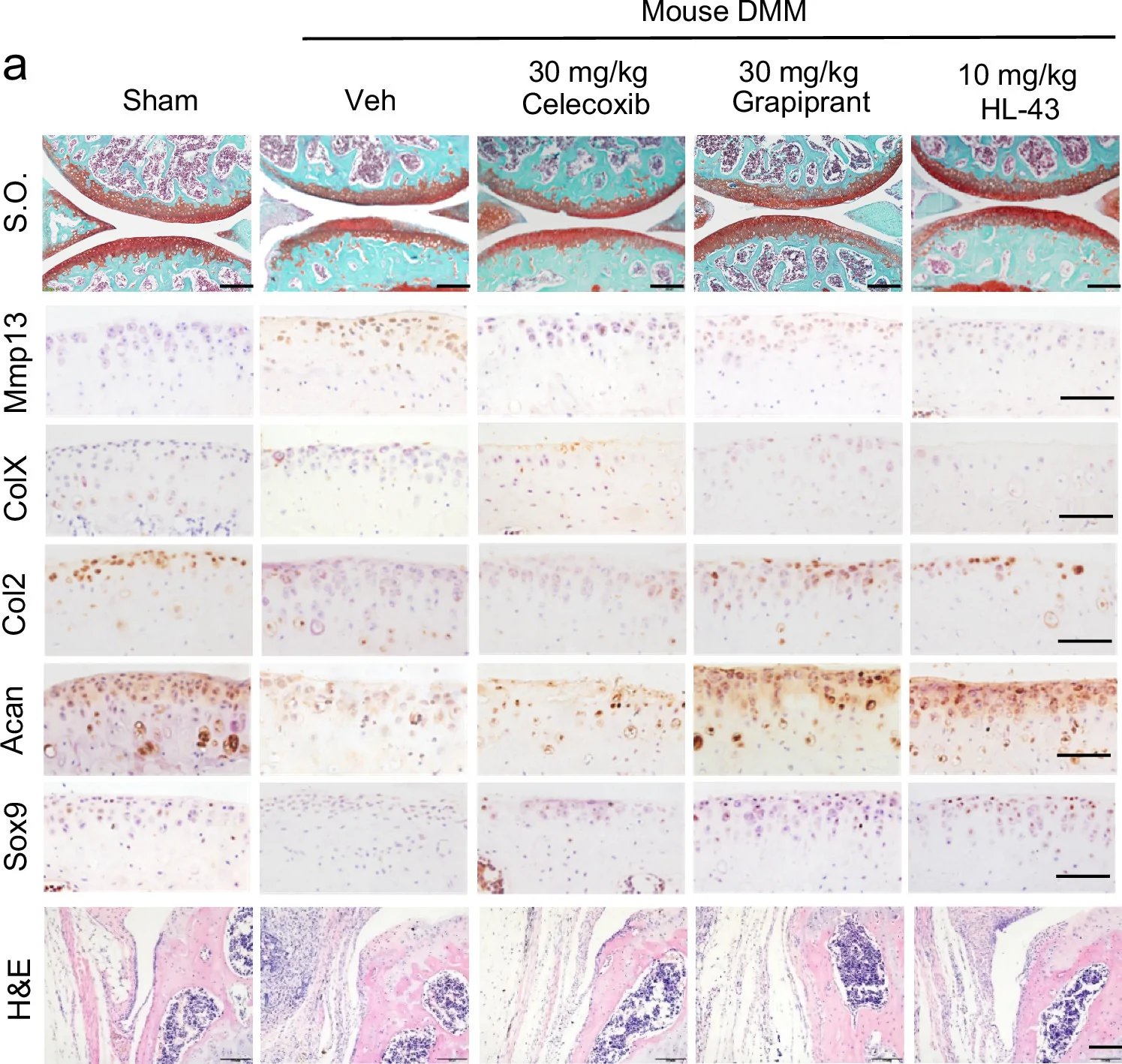

**Correct Supplementary Figure 8**

**Figure:**