# Supplementary material for: A novel prostaglandin E receptor 4 (EP4) small molecule antagonist induces articular cartilage regeneration
Source: Cell Discov. 2022 Mar 8;8:24. doi: 10.1038/s41421-022-00382-6 (PMC8901748; doi:10.1038/s41421-022-00382-6)
Supplement: Supplementary file 1 — Supplemental information [file 41421_2022_382_MOESM1_ESM.pdf]

Gene	Fold change	P value
EP4	2.16	0.0040

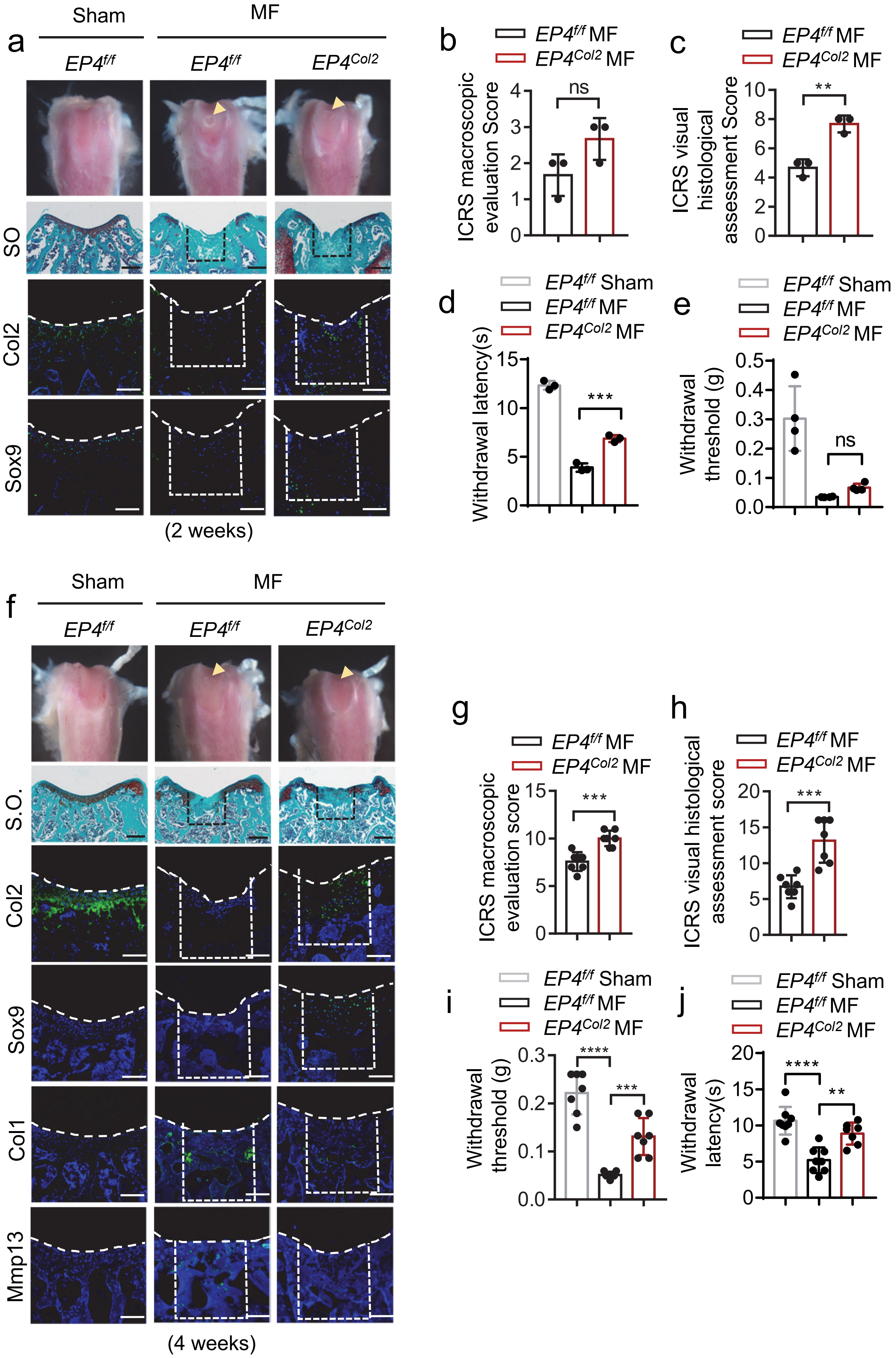

a
Sham
MF
EP4f/f
EP4f/f
EP4Col2
SO
Col2
Sox9
(2 weeks)
b
EP4f/f MF
EP4Col2 MF
ICRS macroscopic evaluation Score
ns
c
ICRS visual histological assessment Score
**
d
EP4f/f Sham
Withdrawal latency(s)
***
e
Withdrawal threshold (g)
ns
f
S.O.
Col1
Mmp13
(4 weeks)
g
ICRS macroscopic evaluation score
***
h
ICRS visual histological assessment score
***
i
Withdrawal threshold (g)
****
***
j
Withdrawal latency(s)
****
**

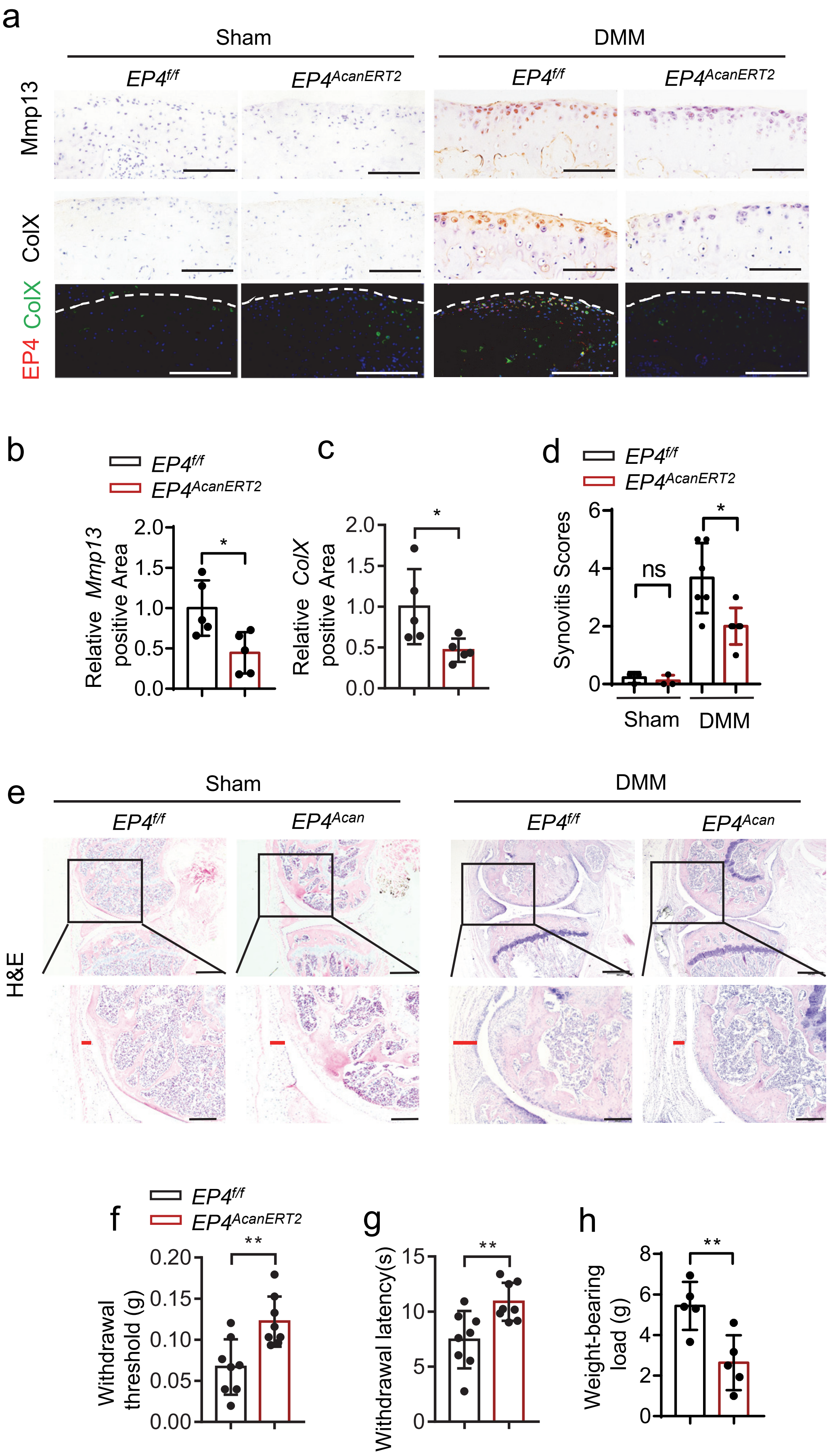
a
Sham
DMM
EP4f/f
EP4AcanERT2
EP4f/f
EP4AcanERT2
Mmp13
ColX
EP4 ColX
b
EP4f/f
EP4AcanERT2
Relative Mmp13 positive Area
2.0
1.5
1.0
0.5
0.0
*
c
Relative ColX positive Area
2.0
1.5
1.0
0.5
0.0
*
d
EP4f/f
EP4AcanERT2
Synovitis Scores
6
4
2
0
ns
*
Sham
DMM
e
Sham
DMM
EP4f/f
EP4Acan
EP4f/f
EP4Acan
H&E
f
EP4f/f
EP4AcanERT2
Withdrawal threshold (g)
0.20
0.15
0.10
0.05
0.00
**
g
Withdrawal latency(s)
15
10
5
0
**
h
Weight-bearing load (g)
8
6
4
2
0
**

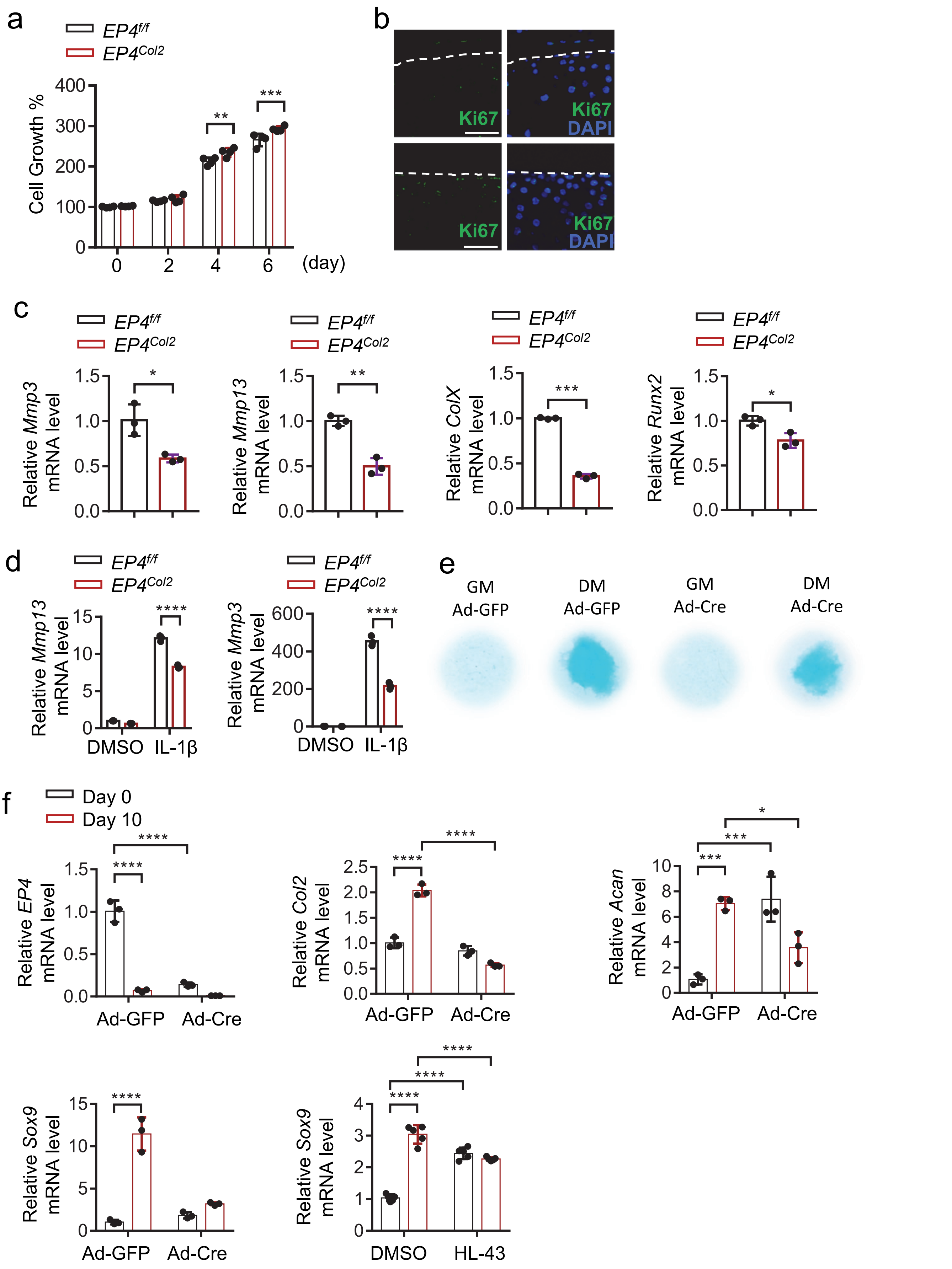
a
EP4f/f
EP4Col2
Cell Growth %
400
300
200
100
0
**
***
0
2
4
6
(day)
b
Ki67
Ki67
DAPI
Ki67
Ki67
DAPI
c
Relative Mmp3 mRNA level
Relative Mmp13 mRNA level
Relative ColX mRNA level
Relative Runx2 mRNA level
1.5
1.0
0.5
0.0
*
**
***
d
Relative Mmp13 mRNA level
15
10
5
0
****
DMSO
IL-1β
Relative Mmp3 mRNA level
600
400
200
0
e
GM
Ad-GFP
DM
Ad-GFP
GM
Ad-Cre
DM
Ad-Cre
f
Day 0
Day 10
Relative EP4 mRNA level
Relative Col2 mRNA level
Relative Acan mRNA level
****
***
Ad-GFP
Ad-Cre
2.5
2.0
10
8
6
4
2
Relative Sox9 mRNA level
HL-43

a

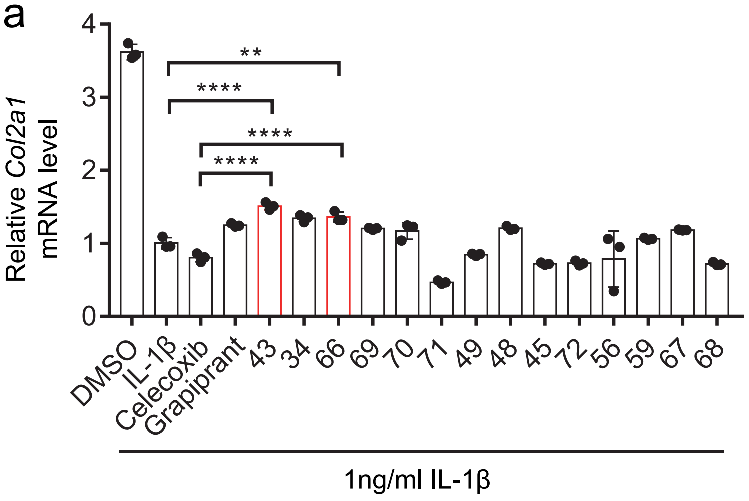

b

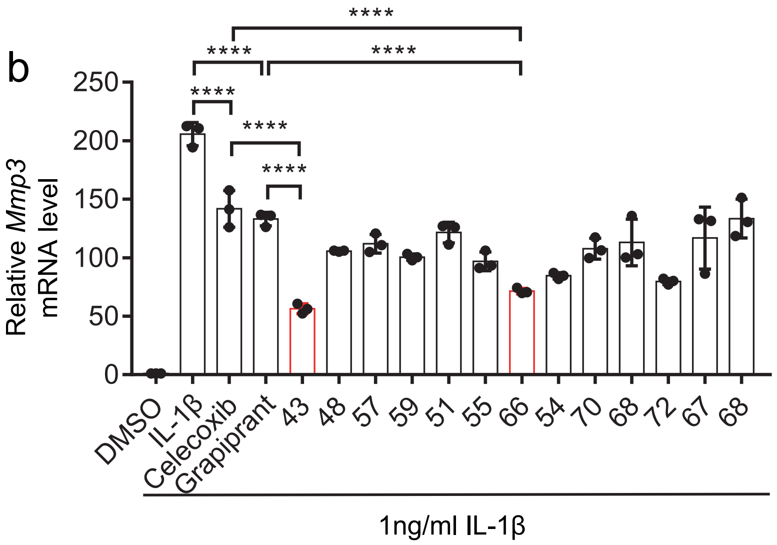

c

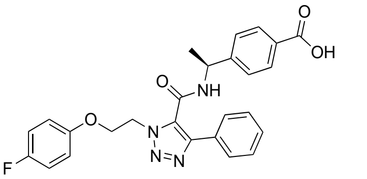

HL-43 (**43**)

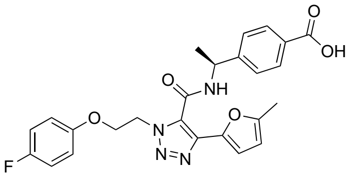

HL-66 (**66**)

d

24hpf	Celecoxib				Grapiprant			HL-43			DMSO	
Concentration (mM)	0.03	0.1	0.3	1	0.3	1	3	0.3	1	3	2µl	12µl
Mortality	0/30	7/30	12/30	18/30	4/30	14/30	13/30	0/30	3/30	6/30	1/30	1/30

72hpf	Celecoxib				Grapiprant			HL-43			DMSO	
Concentration (mM)	0.03	0.1	0.3	1	0.3	1	3	0.3	1	3	2µl	12µl
Hatching	12/30	8/30	13/30	3/30	24/30	16/30	17/30	28/30	26/30	23/30	29/30	28/30
Deformities	4/30	3/30	7/30	2/30	4/30	3/30	4/30	2/30	2/30	4/30	0/30	0/30

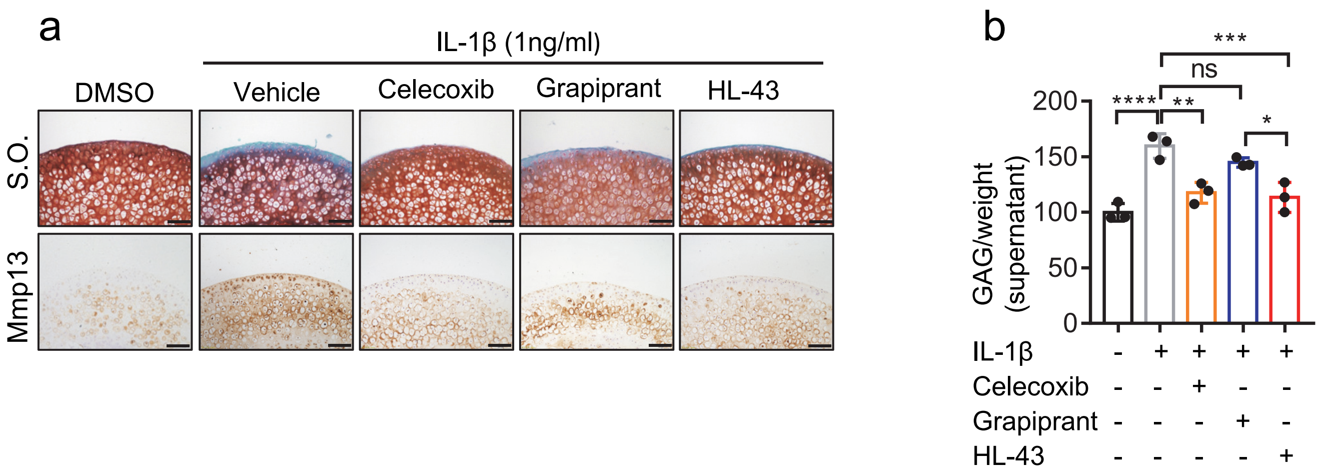
a
IL-1β (1ng/ml)
DMSO
Vehicle
Celecoxib
Grapiprant
HL-43
S.O.
Mmp13
b
GAG/weight
(supernatant)
200
150
100
50
0
****
**
ns
***
*
IL-1β - + + + +
Celecoxib - - + - -
Grapiprant - - - + -
HL-43 - - - - +

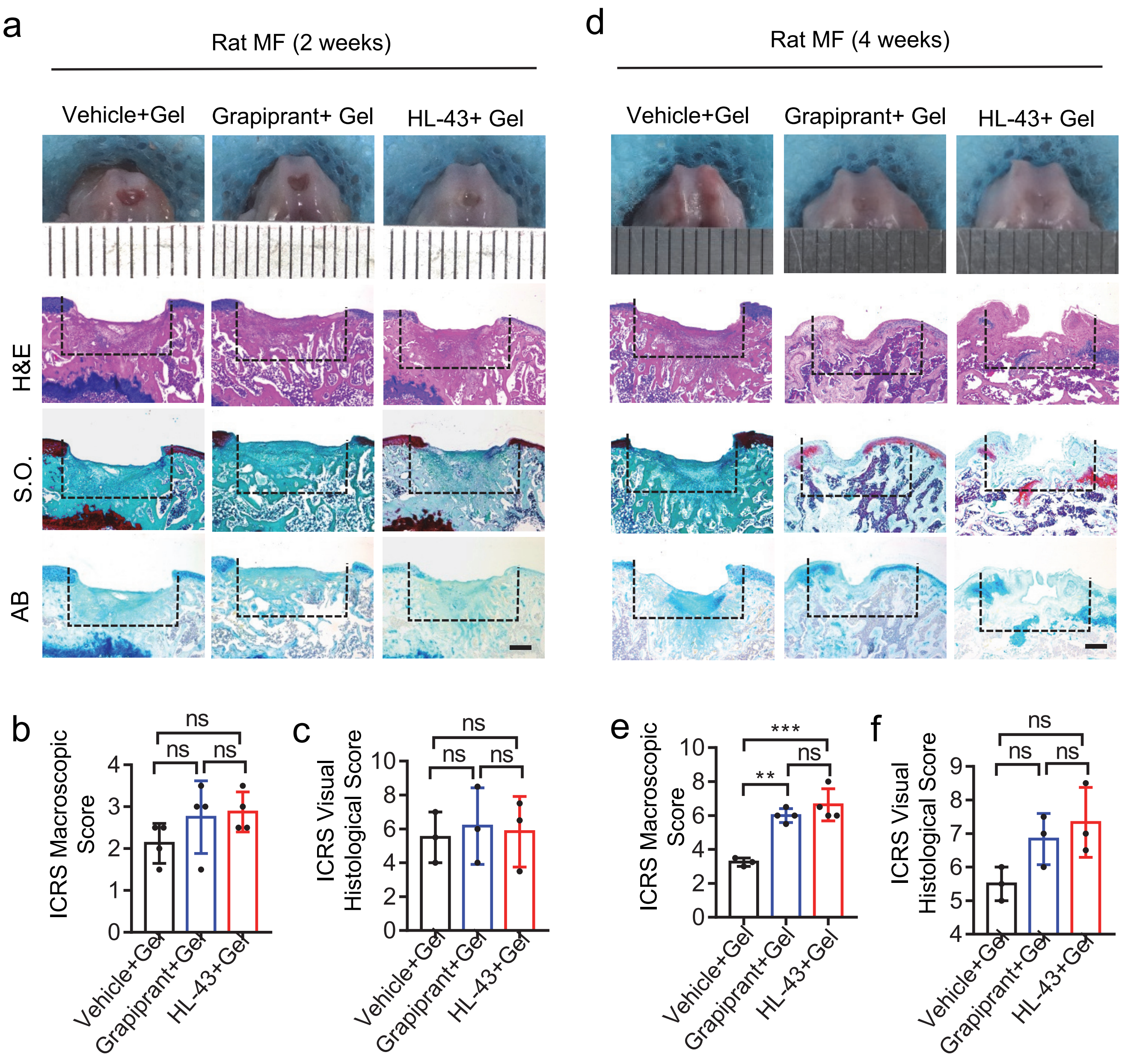
a
Rat MF (2 weeks)
Vehicle+Gel
Grapiprant+ Gel
HL-43+ Gel
H&E
S.O.
AB
d
Rat MF (4 weeks)
Vehicle+Gel
Grapiprant+ Gel
HL-43+ Gel
b
ICRS Macroscopic Score
ns
Vehicle+Gel
Grapiprant+Gel
HL-43+Gel
c
ICRS Visual Histological Score
ns
Vehicle+Gel
Grapiprant+Gel
HL-43+Gel
e
ICRS Macroscopic Score
***
**
ns
Vehicle+Gel
Grapiprant+Gel
HL-43+Gel
f
ICRS Visual Histological Score
ns
Vehicle+Gel
Grapiprant+Gel
HL-43+Gel

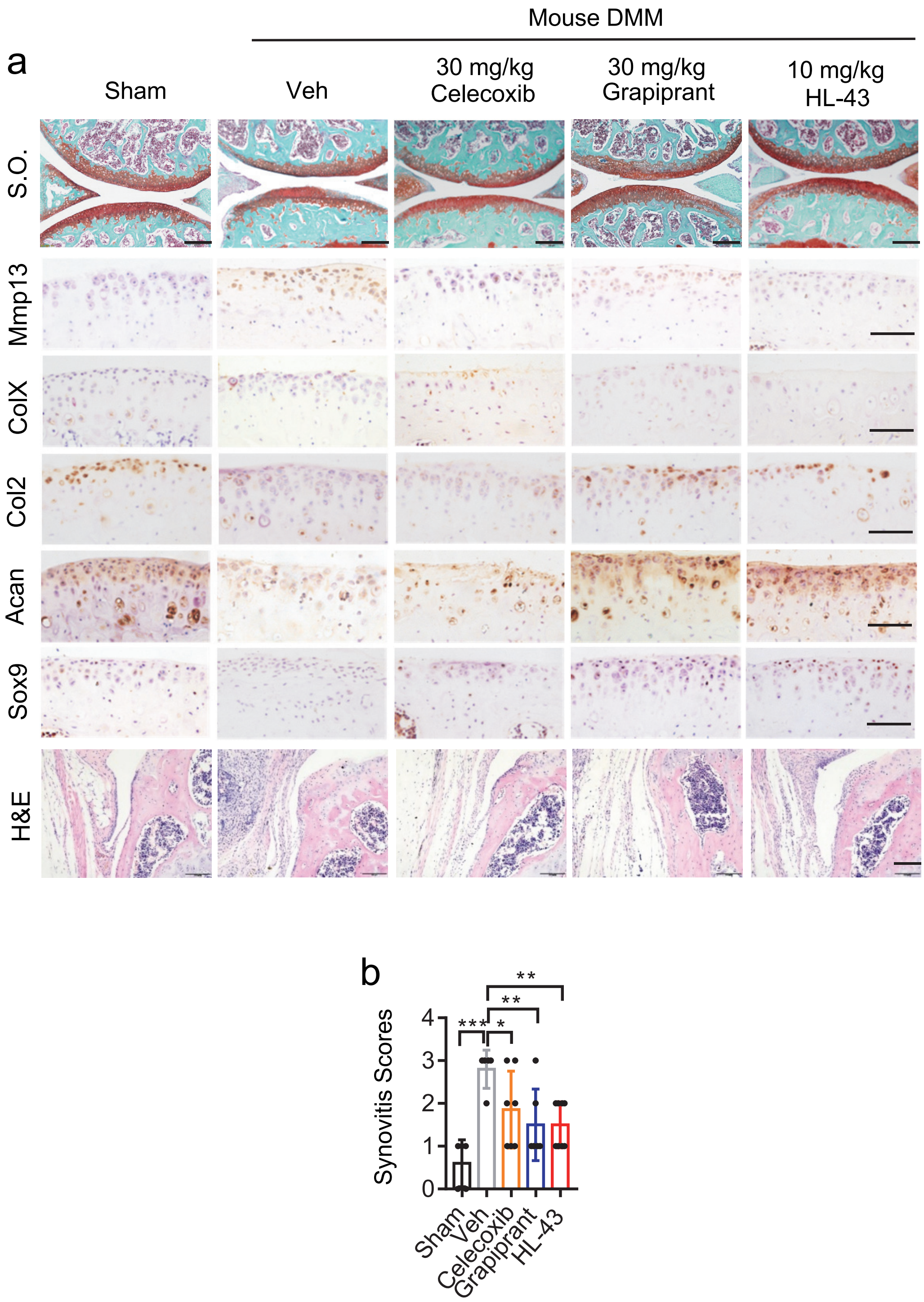
Mouse DMM
a
Sham
Veh
30 mg/kg Celecoxib
30 mg/kg Grapiprant
10 mg/kg HL-43
S.O.
Mmp13
ColX
Col2
Acan
Sox9
H&E
b
Synovitis Scores
0
1
2
3
4
***
*
**
**
Sham
Veh
Celecoxib
Grapiprant
HL-43

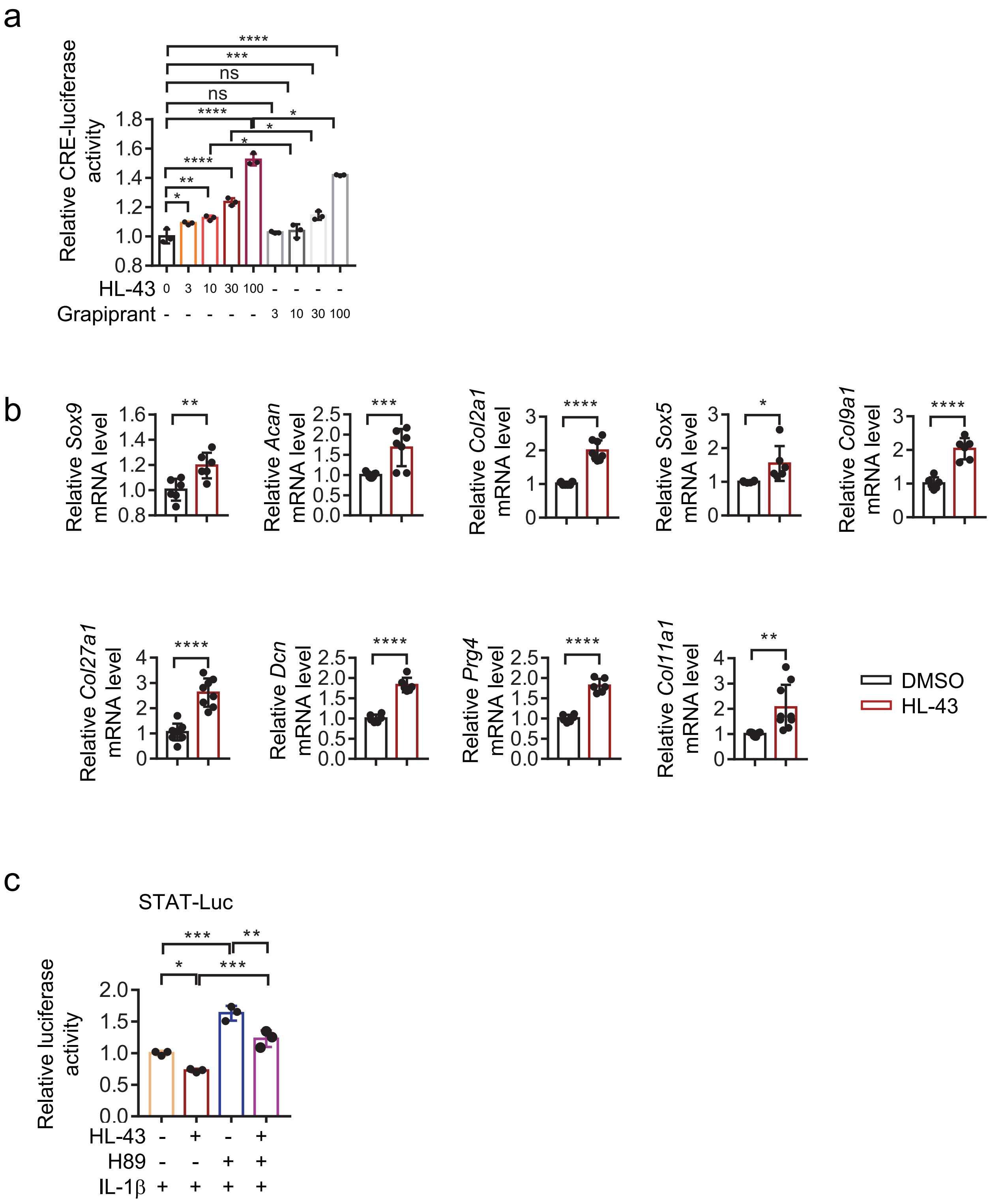
a
Relative CRE-luciferase activity
HL-43 0 3 10 30 100 - - - -
Grapiprant - - - - - 3 10 30 100
b
Relative Sox9 mRNA level
Relative Acan mRNA level
Relative Col2a1 mRNA level
Relative Sox5 mRNA level
Relative Col9a1 mRNA level
Relative Col27a1 mRNA level
Relative Dcn mRNA level
Relative Prg4 mRNA level
Relative Col11a1 mRNA level
DMSO
HL-43
c
STAT-Luc
Relative luciferase activity
HL-43 - + - +
H89 - - + +
IL-1β + + + +

PCR primer sequences

Human *EP4*	Forward primer: AACTTGATGGCTGCGAAGACCTAC
	Reverse primer: TTCTAATATCTGGGCCTCTGCTGTG
Mouse *EP4*	Forward primer: GTGCGGAGATCCAGATGGTC
	Reverse primer: TCACCACGTTTGGCTGATATAAC
Human *MMP13*	Forward primer: TGGTCCAGGAGATGAAGACC
	Reverse primer: TCCTCGGAGACTGGTAATGG
Mouse *Mmp13*	Forward primer: GTGTGGAGTTATGATGATGT
	Reverse primer: TGCGATTACTCCAGATACTG
Human *MMP3*	Forward primer: CACTCACAGACCTGACTCGG
	Reverse primer: GAGTCAGGGGGAGGTCCATA
Mouse *Mmp3*	Forward primer: TTGACTCAAGGGTGGATGCTGTCT
	Reverse primer: GCACATGCTGAACAAAGCACTTCC
Mouse *Adamts5*	Forward primer: TCGCAGATACAGCTGTGGTGAAGA
	Reverse primer: ATTGTGTGTCTCCTTTGTGTAGGCCC
Mouse *Sox9*	Forward primer: CAACTTTGCCAGCTTGCACG
	Reverse primer: CTTAGTCCACCCCTCCTCAC
Mouse *Aggrecan*	Forward primer: GAAGACGACATCACCATCCAG
	Reverse primer: CTGTCTTTGTCACCCACACATG
Mouse *Col2α1*	Forward primer: CCTCCGTCTACTGTCCACTGA
	Reverse primer: ATTGGAGCCCTGGATGAGCA
Mouse *ColX*	Forward primer: ATGCCTTGTTCTCCTCTTACTG
	Reverse primer: TGCTGAACGGTACCAAACG
Mouse *Runx2*	Forward primer: CGTCCACTGTCACTTTAATAGCTC
	Reverse primer: GTAGCCAGGTTCAACGATCTG
Human *GAPDH*	Forward primer: CGTCTTCACCACCATGGAGA
	Reverse primer: CGGCCATCACGCCACAGTTT
Mouse *GAPDH*	Forward primer: AGGTCGGTGTGAACGGATTTG
	Reverse primer: TGTAGACCATGTAGTTGAGGTCA
Human *COL2A1*	Forward primer: CCAGATGACCTTCCTACGCC
	Reverse primer: TTCAGGGCAGTGTACGTGAAC
Human *ACAN*	Forward primer: AGTCCTCAAGCCTCCTGTACTCA
	Reverse primer: GCAGTTGATTCTGATTCACGTTTC
Human *SOX9*	Forward primer: GCTCTGGAGACTTCTGAACGA
	Reverse primer: CCGTTCTTCACCGACTTCCT
Mouse *Mmp9*	Forward primer: ACCACATCGAACTTCGA
	Reverse primer: CGACCATACAGATACTG
Mouse *Sox5*	Forward primer: GCTTCAGCAACAGATCCAGG
	Reverse primer: AAGGAGGAATCCTTGCTGGG
Mouse *Col9a1*	Forward primer: CTGGTGTACCTGGGTCTCGT
	Reverse primer: TTCTCCTTTCGCCCCTTTAT
Mouse *Col27a1*	Forward primer: CCTTCCCGTAGGGACTCCAT
	Reverse primer: GGCACAGTAATTGTGAGCGAC
Mouse *Dcn*	Forward primer: AGGCTTCCTACTCGGCTGTGA
	Reverse primer: GTTCGGCGGCATTTGACTTT
Mouse *Prg4*	Forward primer: TTTTGGCCGGGAGACTCAATC
	Reverse primer: CAGCGTAGTCAGTCCATCCAC
Mouse *Col11a1*	Forward primer: GCCCGGCAAAACTTCACCTACAA
	Reverse primer: TCGAGACGCACAGCCATCATACAA

Supplementary Figure Legends

Supplementary Figure S1. EP4 is upregulated in IL-1β treated mouse articular chondrocytes

The expression of *EP4* in mouse primary chondrocytes treated with or without IL-1β is analyzed from microarray analysis data GSE104793.

Supplementary Figure S2 Cartilage-specific deletion of *EP4* promotes cartilage regeneration after MF surgery (refer to Fig. 2a-2e).

Representative images of distal femur of Sham-operation or MF at 2 weeks after surgery in *EP4*$^{f/f}$ or *Ep4*$^{f/f}$; *Col2-Cre* (*Ep4*^{Col2}) mice (a). Yellow arrows show the defect region. The Safranin-O/Fast Green (S.O.) staining of the articular cartilage sections. Scale bar, 200 μm. The articular cartilage sections from sham or MF surgery were stained with indicated antibodies. Nucleus were shown by DAPI staining. Scale bars, 100 μm. Dotted lines indicated the surface of articular cartilage and dotted boxes indicated the defect region. (b) International Cartilage Repair Society (ICRS) macroscopic evaluation score assessment was performed. (c) ICRS Visual Histological Score of regenerated tissues were performed. n=3 for each group, error bars are mean ±s.d., *P < 0.05, **P < 0.01 by student-t test. IF, immunofluorescence. ns, not significant.

The MF surgery-induced pain of the indicated groups (refer to Supplementary Fig. S2a-S2c) was measured by (d) radiant heat paw-withdrawal test, and (e) Von Frey assay. Error bars are mean ±s.d., n=3 for each group, ***P < 0.001 by one-way ANOVA with

multiple comparison using Tukey method, ns, not significant.

Representative images of distal femur of Sham-operation or MF at 4 weeks after surgery in *EP4^f/f^* or *Ep4^f/f^*; *Col2-Cre* (*Ep4^Col2^*) mice (f). Yellow arrows show the defect region. The Safranin-O/Fast Green (S.O.) staining of the articular cartilage sections. Scale bar, 200 μm. The articular cartilage sections from sham or MF surgery were stained with indicated antibodies. Nucleus were shown by DAPI staining. Scale bars, 100 μm. Dotted lines indicated the surface of articular cartilage and dotted boxes indicated the defect region. (g) International Cartilage Repair Society (ICRS) macroscopic evaluation score assessment was performed. (h) ICRS Visual Histological Score of regenerated tissues were performed. n=7 for 4 weeks group. Error bars are mean ±s.d., ***P < 0.001 by student-t test. IF, immunofluorescence.

The MF surgery-induced pain 4 weeks after the surgery was measured by Von Frey assay (i), and radiant heat paw-withdrawal test (j). Error bars are mean ±s.d., n=7 for each group, **P < 0.01, ***P < 0.001, ****P < 0.0001 by one-way ANOVA with multiple comparison using Tukey method.

Supplementary Figure S3. Cartilage-specific deletion of *EP4* inhibits cartilage catabolism and synovitis.

(a) Representative images of immunohistochemistry staining and immunofluorescence staining of Sham-operation or DMM at 8 weeks after surgery in *EP4^f/f^* or *Ep4^AcanERT2^* mice (refer to Fig. 2g). Scale bar, 200 μm. The Mmp13 and ColX expression were quantified in (b) and (c), respectively. Error bars are mean ±s.d., n=5 for each group,

*P < 0.05 by student-t test.

(d) Synovitis score for each group, and (e) the corresponding representative images of H&E staining. Error bars are mean ±s.d., n=3 for Sham groups, n=6 for DMM groups, *P < 0.05 by ordinary one-way ANOVA with multiple comparison using Tukey method. Top panel scale bar, 500 μm; bottom panel scale bar, 200 μm. ns, not significant. Red lines indicate synovial thickness of the knee joints.

The DMM surgery-induced pain for the indicated groups (refer to Fig. 2g) was measured by (f) Von Frey assay, (g) radiant heat paw-withdrawal test, and (h) hind limb weight bearing analysis. Error bars are mean ±s.d., n=8 in (f) and (g), n=5 in (h), **P < 0.01 by student-t test.

Supplementary Figure S4. Cartilage-specific deletion of EP4 mildly induces chondrocyte proliferation and attenuates chondrocyte catabolism.

(a) Cell growth was assessed by Sulphorhodamine B (SRB) assay. Primary articular chondrocytes were isolated from $Ep4^{Col2}$ and $Ep4^{f/f}$ knee joints of new born pups. Cells were seeded at 2,000/ well in 96-cluster well culture plates, following 0, 2, 4, 6 days incubation period in DMEM/F12 medium. The experiments were repeated in triplicate. Error bars are mean ±s.d., n=4 for each group, **P < 0.01, ***P < 0.001, by two-way ANOVA with multiple comparison using Sidak method.

(b) Ki67 staining of articular cartilage of $Ep4^{Col2}$ knee joints and the control $Ep4^{f/f}$ in one-month-old male mice. Scale bars are 100 μm. Dotted lines indicated the surface of articular cartilage.

(c) qRT-PCR analyses of *Mmp3*, *Mmp13*, *ColX* and *Runx2* expression in primary articular chondrocytes of $EP4^{f/f}$ and $Ep4^{Col2}$, 7 days after chondrogenic differentiation. Error bars are mean ±s.d., *P < 0.05, ***P < 0.001, by student-t test.

(d) qRT-PCR analyses of *Mmp13* and *Mmp3* expression in primary articular chondrocytes from $EP4^{f/f}$ and $Ep4^{Col2}$mice treated with IL-1β for 24 hours. Error bars are mean ±s.d., n=3, ****P < 0.0001 by one-way ANOVA with multiple comparison using Tukey method.

(e) BMSC were isolated from 8 weeks old $Ep4^{f/f}$ mice. Micromass of the BMSCs cultured in the chondrogenic media for 16 days were stained with Alcian blue after the indicated treatment. GM, growth medium. DM, differentiation medium.

(f) qRT-PCR analyses of *EP4*, *Col2*, *Acan*, and *Sox9* expression in BMSC of $EP4^{f/f}$ treated with Ad-GFP, Ad-Cre, or treated with DMSO and HL-43 (30 μM), 10 days after chondrogenic differentiation. GM, growth medium. DM, differentiation medium. Error bars are mean ±s.d., *P < 0.05, ***P < 0.001, ****P < 0.0001 by two-way ANOVA with multiple comparison using Tukey method.

Supplementary Figure S5. Identification of a novel EP4 antagonist with low toxicity

(a) Screening EP4 antagonists for effects on cartilage anabolism. qRT-PCR analysis of *Col2a1* expressed in primary mouse chondrocytes treated with EP4 antagonists (2 nM) and 1ng/ml IL-1β for 24 hr (n=3).

(b) Screening EP4 antagonists for effects on cartilage catabolism. qRT-PCR analysis of

Mmp3 expressed in primary mouse chondrocytes treated with EP4 antagonists (30 μM) and 1ng/ml IL-1β for 24 hr (n=3). Error bars are mean ±s.d., n=3, **P < 0.01, ****P < 0.0001 by one-way ANOVA with multiple comparison using Tukey method.

(c) Chemical structure of EP4 antagonists HL-43 and HL-66.

(d) Mortality rates of the zebrafish embryos after 24 hr. Hatching and deformity rates of the larvae after 72 hpf. Embryos were treated with the indicated concentration compounds (n=30).

Supplementary Figure S6. EP4 antagonist HL-43 inhibited cartilage catabolism

(a) Mouse femoral heads was treated with or without 1ng/ml IL-1β and indicated chemical compounds for 48 hr. Representative images of immunohistochemistry staining of Mmp13 in each group. Scale bars, 100 μm. (b). Release of total GAG into the media of mouse femoral heads was measured by DMMB assays, and normalized to the wet weight of the cartilage explant and then normalized to vehicle to calculate the release of GAG. Error bars are mean±s.d., n = 3 for each group. *P < 0.05, **P < 0.01, ***P < 0.001, ****P < 0.0001 by one-way ANOVA with multiple comparison using the Dunnett method. ns, not significant.

Supplementary Figure S7. HL-43 promoted cartilage regeneration in MF surgery-induced CD rat model

EP4 antagonists regulated cartilage regeneration in rats at 2 weeks (a-c) and 4 weeks (d-f) after MF surgery. Macroscopic appearance, H&E staining, Safranin-O/Fast Green

(S.O.) staining, and Alcian blue (AB) staining of the articular cartilage. Black dotted boxes indicated the defect region. Scale bar = 500 μm. The International Cartilage Repair Society (ICRS) macroscopic score of regenerated tissues analysis was performed (b and e). n=4 for each group. ICRS Visual Histological Score of regenerated tissues analysis was performed (c and f). n=3 for each group. Error bars are mean ±s.d., **P < 0.01, ***P < 0.001, by one-way ANOVA with multiple comparison using Tukey method. ns, not significant.

Supplementary Figure S8. EP4 antagonists enhanced cartilage repair and inhibited joint synovitis in the DMM surgery-induced experimental OA mouse model (refer to Fig. 6e-6g).

(a) EP4 antagonist HL-43 enhanced cartilage repair by upregulating cartilage anabolism and reducing cartilage catabolism in DMM surgery-induced experimental OA mouse model. Safranin-O/Fast-green (S.O.) and H&E staining of the knee joints 6 weeks after DMM surgery. Celecoxib, Grapiprant at 30 mg/kg, HL-43 at 10 mg/kg daily by gavage. Scale bar, 200 μm. Immunohistochemistry of Mmp13, ColX, Col2, Acan, Sox9 in knee joint sections from each group. Scale bar, 100 μm.

(b) Synovitis score of each group. Error bars are mean ±s.d., $n \geqslant 5$ for each group, *P < 0.05, ***P < 0.001 by one-way ANOVA with multiple comparison using Dunnett method.

Supplementary Figure S9. EP4 antagonist HL-43 upregulated the anabolic

makers and had better efficiency than Grapiprant

(a) HL-43 and Grapiprant activated the CRE-Luc reporter. ATDC5 cells were transfected with the CRE-Luc reporter and treated with the indicated concentrations (μM) of HL-43 or Grapiprant for 12 hr, the cell lysates were subjected to a Dual-Luciferase assays (n=3). Error bars are mean ±s.d., *P < 0.05, **P < 0.01, ***P < 0.001, ****P < 0.0001 by one-way ANOVA with multiple comparison using Tukey method.

(b) qRT-PCR analysis of *Sox9, Acan, Col2a1, Sox5, Col9a1, Col27a1, Dcn, Prg4, Col11a1* expression in ATDC5 cells that treated with DMSO or 30μM HL-43 for 24 hr were performed. (n⩾6).

(c) PKA inhibitor H89 could not abolish HL-43 induced decrease of STAT luciferase reporter activity. After transfecting with the STAT-Luc reporter and HA-STAT3, the ATDC5 cells were treated with 2ng/ml IL-1β, 30μM HL-43, or 10 μM H89 for 12 hr. The cell lysates were subjected to Dual-Luciferase assays (n=3). Error bars are mean ±s.d., *P < 0.05, **P < 0.01, ***P < 0.001, ****P < 0.0001 by two-way ANOVA with multiple comparison using Tukey method.
